# Targeting Oxidative Stress and Apoptosis via PI3K/Akt/Nrf2 Pathway: The Therapeutic Role of *Bletilla striata* Polysaccharide in Diabetic Wound Repair

**DOI:** 10.1155/jdr/5751331

**Published:** 2026-01-28

**Authors:** Shuangyi Xu, Zerui Ni, Tong Zhang, Xiaowei Zhang, Xiaomei Li, Limin Bai, Lu Yu, Gang Xu

**Affiliations:** ^1^ Department of Plastic and Burn Surgery, Northern Jiangsu People′s Hospital, Yangzhou, China, yzsbh.com; ^2^ Yangzhou University, Yangzhou, China, yzu.edu.cn

**Keywords:** antiapoptosis, antioxidant, *Bletilla striata* polysaccharide, diabetic wound, high glucose

## Abstract

**Background:**

Diabetic wounds are challenging and lack efficient therapeutic solutions. *Bletilla striata* polysaccharide (BSP) has garnered interest for its bioactivity and antioxidant ability in wound healing. This research explores the mechanisms through which BSP alleviates oxidative stress (OS) in L929 cells and prevents cell apoptosis under high‐glucose (HG) conditions. Furthermore, the research evaluates its promise as a novel therapeutic approach for facilitating recovery in diabetic wounds.

**Methods:**

Various doses of glucose and BSP were administered to L929 cells to evaluate their effects on cell viability, OS, activation of the PI3K/Akt/Nrf2 signaling pathway, and apoptosis. These effects were assessed using CCK‐8 assays, commercial kits, and western blots (WBs). For in vivo validation in diabetic mice with skin wounds, Masson′s trichrome staining, hematoxylin and eosin (H&E) staining, and WB were employed. Additionally, inhibitors of the PI3K/Akt/Nrf2 signaling pathway were used in both in vitro and in vivo experiments.

**Results:**

In vitro, L929 cells exposed to HG stimuli exhibited OS and apoptosis. However, BSP mitigated these effects by promoting Nrf2 nuclear translocation through the phosphorylation of PI3K and Akt. In diabetic mice, BSP treatment enhanced wound healing rates compared to the control in vivo. This improvement was clear from a substantial reduction in wound areas, decreased inflammation, robust collagen deposition, and extensive reepithelization, which resulted from the inhibition of the intrinsic apoptosis process mediated through the activation of the PI3K/Akt/Nrf2 signaling pathway.

**Conclusion:**

Our research emphasizes that BSP serves as a potential therapeutic resolution targeting diabetic wounds for its excellent OS‐relieving and antiapoptosis properties. Our findings reveal the value of natural polysaccharides in the treatment of the complications of diabetes and indicate that BSP promotes the healing of diabetic wounds via the PI3K/Akt/Nrf2 signaling pathway.

## 1. Introduction

Diabetes mellitus (DM) represents a prevalent metabolic disorder frequently encountered in clinical settings, often giving rise to various severe health complications. Among these, diabetic wounds stand out as a particularly serious chronic condition, driven by prolonged exposure to high‐glucose (HG) environments. These wounds are clinically defined by delayed, dysregulated healing processes and can progress to limb amputation, systemic infections, or mortality. Current therapeutic strategies remain limited and largely inadequate. Standard clinical protocols emphasize foundational wound management—including debridement, selection of appropriate wound dressings, infection prevention, and pressure relief. However, there is a notable scarcity of pharmacological or bioactive agents designed specifically for diabetic wounds. Existing interventions frequently fall short in comprehensively addressing the multifactorial pathology underlying impaired wound healing in diabetes, which involves impaired angiogenesis, increased inflammation, hypoxia, and dysfunctions at the cellular level [1–3]. Therefore, it is imperative to develop new, effective diabetic wound healing therapies.

As the predominant cell type of the dermis, skin fibroblasts are the key role in the healing of diabetic wounds. They are not only involved in regulating local inflammatory responses, collagen production and deposition, angiogenesis, and extracellular matrix reorganization but also contribute significantly to the maintenance of tissue homeostasis [4, 5]. In diabetic wounds, the endogenous redox balance is disrupted, as evidenced by decreased activities of glutathione (GSH), superoxide dismutase (SOD), and catalase (CAT). This leads to oxidative stress (OS) and further triggers signaling pathways associated with apoptosis. Additionally, the OS of the HG microenvironment also further inhibits the migration of fibroblasts and leads to apoptosis [6–8]. Therefore, targeting OS and apoptosis in skin fibroblasts under HG conditions offers an effective approach for treating diabetic wounds.

BSP (*Bletilla striata* polysaccharide) is a natural polysaccharide extracted from the rhizome of *Bletilla striata*, a plant long used in traditional Chinese medicine. In recent years, it has gained increasing interest in modern medical research owing to a range of documented pharmacological effects, such as anti‐inflammatory, antioxidant, wound healing, antitumor, antifibrotic, and immunomodulatory activities, which have been observed in vitro [9–12]. Additionally, BSP can serve as an effective carrier for active components in pharmaceutical formulations, such as microneedles, freeze‐dried wafers, and gels [13–15]. Recent studies indicate its promising application in diabetic wound repair. For example, Zhao et al. reported that BSP promotes wound closure in a diabetic mouse model by inhibiting the NLRP3 inflammasome, which alleviates local inflammation and enhances tissue regeneration [16]. Although these findings reveal the anti‐inflammatory properties of BSP in diabetic wounds, its influence on OS and fibroblast apoptosis requires further elucidation. In particular, the potential involvement of BSP in regulating the PI3K/Akt/Nrf2 pathway, a critical signaling cascade for cellular antioxidant defense and survival under HG stress, remains unexplored.

The PI3K/Akt signaling cascade plays a fundamental role in modulating essential cellular processes including migration, proliferation, programmed cell death, and metabolic homeostasis [17]. Accumulating evidence indicates that pharmacological or genetic activation of this pathway can significantly decrease apoptotic cell numbers in diabetic wounds and facilitate the healing process [18]. Meanwhile, nuclear factor erythroid 2‐related factor 2 (Nrf2), a central transcription factor involved in OS response, has been recognized as a critical mediator in diabetic wound repair [19]. Upon activation, Nrf2 induces the transcription of key antioxidant enzymes—including SOD, GSH, and CAT, thereby enhancing reactive oxygen species (ROS) clearance, alleviating oxidative injury, and attenuating cellular apoptosis [20]. In this investigation, we focus on elucidating the protective mechanisms of BSP in L929 fibroblasts under HG settings, specifically examining its impact on the PI3K/Akt/Nrf2 signaling pathway. Our objective is to systematically explore how BSP‐triggered activation of this pathway reduces oxidative damage, inhibits apoptosis, and ultimately improves wound repair outcomes in a diabetic context.

## 2. Materials and Methods

### 2.1. Materials

Mouse fibroblasts (L929 cell line) were got from the Cell Bank of the Chinese Academy of Sciences (Shanghai, China). Dulbecco′s modified Eagle′s medium (DMEM), fetal bovine serum (FBS), penicillin, streptomycin, trypsin, DMSO, bicinchoninic acid (BCA), GSH, SOD, CAT, and MDA assay kits were obtained from Solarbio Industrial Inc. (Shanghai, China). BSP (Product Number: S27914, 70%) was obtained from Shanghai Yuanye Bio‐Technology Co. Ltd. (Shanghai, China). Streptozocin (STZ) was obtained from Anhui Kuer Bioengineering Co. Ltd. (Anhui, China). LY294002, MK‐2206, and ML385 were obtained from MedChemExpress (New Jersey, United States). Primary antibodies against PI3K (Catalog Number: MA5‐32208), p‐PI3K (Catalog Number: MA5‐36955), Akt (Catalog Number: MA5‐14916), p‐Akt (Catalog Number: 44‐621G), Nrf2 (Catalog Number: 710574), Caspase‐3 (Catalog Number: 700182), Caspase‐9 (Catalog Number: MA5‐33121), Bax (Catalog Number: MA5‐14003), Bcl‐XL (Catalog Number: MA5‐15142), Bcl‐2 (Catalog Number: MA5‐11757), GAPDH (Catalog Number: MA5‐15738), *β*‐tubulin (Catalog Number: MA5‐16308), Histone H3 (Catalog Number: 701517), and the secondary antibody: horseradish peroxidase (HRP)–conjugated antibody (Catalog Number: 31430) were purchased from Thermo Fisher Scientific Inc. Sixty healthy ICR mice (20 ± 5 g, 4 weeks old) were purchased from the Institute of Comparative Medicine of Yangzhou University. Mice were housed in individually ventilated cages (22°C, 40%–60% relative humidity, 12 h light cycle) with autoclaved aspen bedding and are free to irradiated chow and water. Three to five mice per cage were acclimated for 7 days. This study was approved by the Ethics Committee for Laboratory Animals of Yangzhou University (No. 202506035) and performed following the guidelines of Jiangsu Laboratory Animal Welfare and Ethics of Jiangsu Administrative Committee of Laboratory Animals.

### 2.2. Culture of L929 Cells and Cell Grouping

For cell resuscitation, the cryovial was first rapidly thawed using a 37°C water bath. The resulting cell suspension was subsequently transferred into a centrifuge tube and spun at 1000 rpm for 5 min. After discarding the supernatant, the collected cell pellet was resuspended in prewarmed DMEM supplemented with 10% FBS and 1% penicillin–streptomycin solution. Cells were cultured at 37°C in a humidified atmosphere containing 5% CO_2_. When the cell confluence reached 80%–90%, the cells were passaged. L929 cells from Passages 3–6 were used for experiments. The BSP powder was dissolved in DMEM and diluted to 0.25, 0.5, and 1 mg/mL. Firstly, the control group was cultured in the medium at a concentration of 5.5 mM glucose; the glucose gradient groups were cultured in the medium (10, 20, and 30 mM glucose, respectively). Then, a control group, HG group, low‐BSP (L‐BSP) group, medium‐BSP (M‐BSP) group, and high‐BSP (H‐BSP) group were set up; the control group was grown in 5.5 mM glucose medium, and the HG group was grown in 30 mM glucose medium. The L‐BSP, M‐BSP, and H‐BSP groups were pretreated with 0.25, 0.5, and 1 mg/mL BSP 0 for 12 h and then treated the same as the HG group. Furthermore, an HG group, H‐BSP group, H‐BSP + LY294002 group, H‐BSP + MK‐2206 group, and H‐BSP + ML385 group were set up; the HG group and the H‐BSP group were treated the same as mentioned above, and the H‐BSP + LY294002 group, H‐BSP + MK‐2206 group, and H‐BSP + ML385 group were pretreated with BSP at the concentration of 1 mg/mL along with 10 *μ*M LY294002, 1 *μ*M MK‐2206, and 10 *μ*M ML385, respectively, for 12 h, then treated the same as the HG group. Apart from the control group, the glucose gradient group, and the HG group, the others were cultured in medium with BSP and inhibitors, respectively. Afterwards, all groups were later cultured in each concentration of glucose for 48 h.

### 2.3. Cell Viability Assessment

L929 fibroblasts were plated in 96‐well plates at 1 × 10^4^ cells per well and grown in complete DMEM until reaching roughly 80% confluency. Following treatment under the various experimental conditions outlined in Section 2.2, 10 *μ*L of CCK‐8 reagent was introduced into each well. The plates were subsequently incubated for 2 h at 37°C in a 5% CO_2_ atmosphere. Absorbance readings were taken at 450 nm, and the resulting values were used to calculate cell viability. All assays were performed in triplicate.

### 2.4. OS Assays

L929 fibroblasts were plated in 12‐well culture plates at a concentration of 2 × 10^5^ cells per well and maintained in complete DMEM until attaining 80% confluence. Cells were subsequently subjected to the experimental treatments detailed in Section 2.2. Following the intervention, concentrations of MDA, GSH, SOD, and CAT were determined using corresponding commercial assay kits according to the manufacturers′ protocols. All measurements were conducted with three independent replicates.

### 2.5. Construction and Treatment of Diabetic Mouse Wound Model

Four‐week‐old male ICR mice were subjected to a diet rich in sugars and fats for a duration of 4 weeks. Subsequently, they received daily intraperitoneal (IP) injections of STZ (30 mg/kg) for 4 consecutive days. Those exhibiting fasting blood glucose measurements exceeding 11.1 mmol/L on two separate occasions were classified as diabetic members of the study group.

In order to produce full‐thickness excisional wounds in the mice, they were first put under anesthesia through an IP shot of 2% pentobarbital sodium at a dosage of 45 mg/kg. Next, the hair on the backs of the mice was shaved off with an electric razor, and the surgical area was sanitized with Betadine. Following this, full‐thickness excisional wounds were carefully made in the mice′s dorsal skin using modified 10 mm biopsy punches. The date of the wound creation was marked as Day 0.

The mice were randomly divided into the saline group, L‐BSP group, M‐BSP group, H‐BSP group, H‐BSP + LY294002 group, H‐BSP + MK‐2206 group, and H‐BSP + ML385 group, with six mice in each group.

Experiment 1: The mice were separated into four groups: The saline group was given an equal dose of normal saline. The L‐BSP group, M‐BSP group, and H‐BSP group were subcutaneously injected at the wound edges (four sites) with 12.5, 25, and 50 mg/kg BSP (dissolved in normal saline) on Days 0, 3, and 7, respectively.

Experiment 2: The mice were divided into five groups: saline group, H‐BSP group, H‐BSP + LY294002 group, H‐BSP + MK‐2206 group, and H‐BSP + ML385 group. The saline group was given an equal dose of normal saline. The H‐BSP group was subcutaneously injected at the wound edges (four sites) with 50 mg/kg BSP (dissolved in normal saline) on Days 0, 3, and 7. The H‐BSP + LY294002 group and H‐BSP + ML385 group were injected intraperitoneally with 50 mg/kg/day LY294002 and 30 mg/kg/day ML385 daily (both dissolved in DMSO), respectively, followed by subcutaneous injection at the wound edges with 50 mg/kg BSP 1 h later on Days 0, 3, and 7. The H‐BSP + MK‐2206 group received 120 mg/kg/day MK‐2206 via oral gavage daily, in addition to the subcutaneous injection of 50 mg/kg BSP at the wound edges 1 h later on Days 0, 3, and 7.

The injuries were assessed on Days 0, 3, 7, 10, and 14, with photographic documentation captured at each checkpoint. The rate of healing was determined using this formula: Relative wound area percentage (*%*) = (*W* *Δ*/*W*0) × 100*%*, where *W* *Δ* represents the wound size on the measurement day and *W*0 denotes the initial area on Day 0. Upon concluding the study on Day 14, the mice were humanely euthanized via an IP injection of sodium pentobarbital (150 mg/kg) and subsequent cervical dislocation.

### 2.6. Histomorphological Analysis

Two weeks postsurgery, dermal samples from the wound tissue were harvested and submerged in a 4% paraformaldehyde fixative. The specimens were subsequently processed into paraffin‐embedded sections at Solarbio Industrial Inc. (Shanghai, China), where wound healing progression was meticulously evaluated through both H&E and Masson′s trichrome staining techniques.

### 2.7. Western Blot (WB) Analysis

WB was conducted on both cellular and tissue specimens. In cell culture studies, L929 cells were seeded in 12‐well plates at a concentration of 1 × 10^5^ cells per well and subjected to the designated treatments. Cellular proteins were isolated using a lysis buffer with protease and phosphatase inhibitors. For the in vivo component, tissue samples were collected from test subjects and processed with the same extraction buffer to yield total protein lysates. Protein quantification was performed with a BCA assay kit to ensure consistent loading. Equal protein quantities from all samples were resolved on 10% SDS‐PAGE gels and subsequently transferred to PVDF membranes via wet electrophoresis. The membranes were blocked for an hour at room temperature with 5% skim milk in TBST before being incubated overnight at 4°C with primary antibodies targeting PI3K (1:2000), p‐PI3K (1:2000), Akt (1:2000), p‐Akt (1:2000), Nrf2 (1:2000), Caspase‐3 (1:1000), Caspase‐9 (1:1000), Bax (1:2000), Bcl‐XL (1:2000), Bcl‐2 (1:2000), GAPDH (1:10000), *β*‐tubulin (1:5000), and Histone H3 (1:10000). After three 10‐min TBST washes, the membranes were exposed to HRP‐conjugated secondary antibodies for 1 h at room temperature. Following another round of TBST rinses, protein bands were detected using an ECL substrate and captured via chemiluminescent imaging. Band densities were analyzed with ImageJ software and normalized to housekeeping proteins (GAPDH, *β*‐tubulin, or Histone H3) to correct for any loading discrepancies.

### 2.8. Statistical Analysis

Statistical analysis was performed with GraphPad Prism 6, employing Student′s *t*‐test for pairwise comparisons. When evaluating multiple groups, we conducted a two‐way ANOVA followed by Tukey′s test to assess mean differences. Significance thresholds were denoted as follows: ns (not significant) for *p* > 0.05,  ^∗^ *p* < 0.05,  ^∗∗^ *p* < 0.01,  ^∗∗∗^ *p* < 0.001, and  ^∗∗∗∗^ *p* < 0.0001. All data are presented as mean ± standard deviation.

## 3. Results

### 3.1. BSP Alleviates Oxidative Damage and Reduces Apoptosis in L929 Cells Induced by an HG Microenvironment

In diabetic wounds, fibroblasts appear to have a disorder between proliferation and apoptosis. Fibroblasts were exposed to escalating glucose levels to demonstrate the detrimental effects of HG. In Figure S1A–D, MDA levels in the cells substantially increased, concurrently with marked decreases in levels of antioxidant proteins. Compared with the control, L929 cells exposed to glucose exhibited a dose‐dependent upregulation of Caspase‐3, Caspase‐9, and Bax protein expression. Conversely, the antiapoptotic markers Bcl‐XL and Bcl‐2 showed a corresponding decrease in expression levels (Figure S1E–J). The observed changes in protein expression levels imply that the HG microenvironment induces apoptosis in L929 cells. Moreover, glucose administration markedly reduced cell viability relative to untreated controls (Figure S1K). The above findings show that the HG microenvironment could upregulate apoptosis‐related phenotypes and downregulate antiapoptotic phenotypes in L929 cells.

It is also found that the HG microenvironment has the capability to suppress the activation of the PI3K/Akt/Nrf2 pathway. Based on the WB results shown in Figure S2A–D, higher glucose concentrations in L929 cells resulted in a dose‐dependent reduction of PI3K and Akt phosphorylation relative to controls. In addition, cytoplasmic Nrf2 expression increased to varying degrees in experimental groups compared to the control group, while nuclear Nrf2 expression was downregulated conversely (Figure S2E–H). Notably, the group treated with 30 mM glucose reached the highest cytoplasmic Nrf2 expression and lowest nuclear Nrf2 expression.

We pretreated the HG group with BSP to assess the effect of BSP on L929 cells. As illustrated in Figures 1a, 1b, 1c, 1d, and 1e, the H‐BSP group exhibited significantly lower levels of MDA. However, the antioxidant index in the H‐BSP group increased dramatically compared to the HG group, indicating that the cells experienced less OS. Moreover, as illustrated in Figures 1f, 1g, 1h, 1i, 1j, and 1k, BSP treatment led to a marked decrease in Caspase‐3, Caspase‐9, and Bax protein expression levels relative to the HG group. Conversely, Bcl‐2 and Bcl‐XL levels showed a notable upregulation under the same conditions. This shift underscores BSP′s potential role in modulating apoptotic pathways. Additionally, BSP treatment notably enhanced cell survival (Figure 1l). To summarize, BSP can attenuate oxidative damage and relieve apoptosis induced by the HG microenvironment, thereby protecting L929 cells from cell death. Similarly, BSP was also shown to significantly restore the expression of PI3K, Akt, and Nrf2 in L929 cells in the HG microenvironment (Figure S3).

Figure 1The antioxidant and antiapoptosis effects of BSP treatment in L929 cells treated with HG. (a–d) Level of MDA, GSH, SOD, and CAT of L929 cells. (e) WB images. (f–k) Quantitative analysis of WB. (l) Cell viability of L929 cells. HG: high glucose, BSP: *Bletilla striata* polysaccharide, MDA: malondialdehyde, GSH: glutathione, SOD: superoxide dismutase, CAT: catalase. Data are expressed as the mean ± SD (*n* = 3).  ^∗^ *p* < 0.05,  ^∗∗^ *p* < 0.01, and  ^∗∗∗^ *p* < 0.001 versus the indicated groups. ns, no significant difference.

**Figure:**
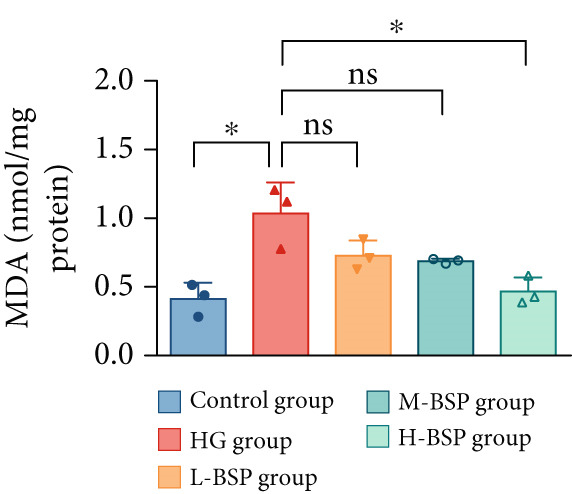
(a)

**Figure:**
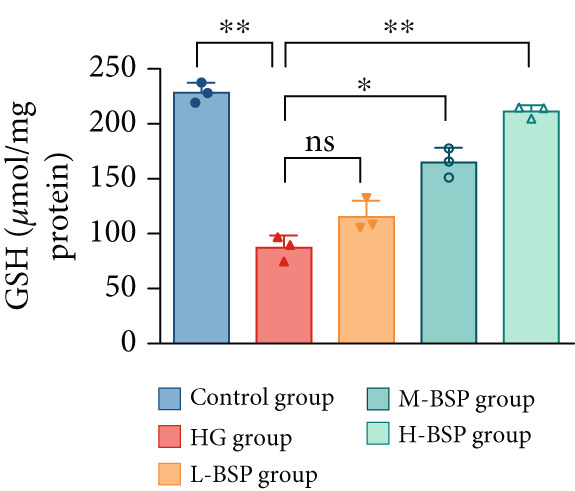
(b)

**Figure:**
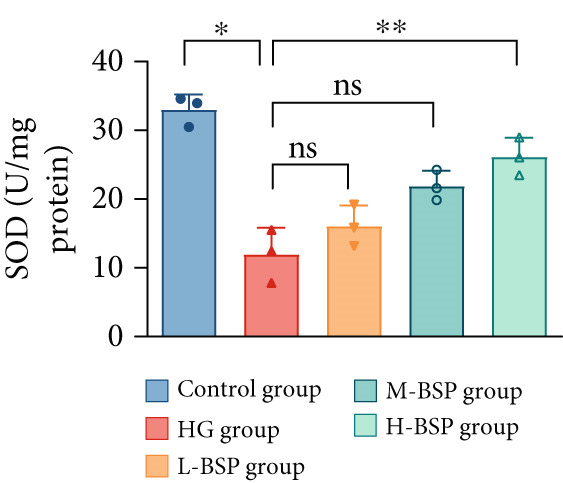
(c)

**Figure:**
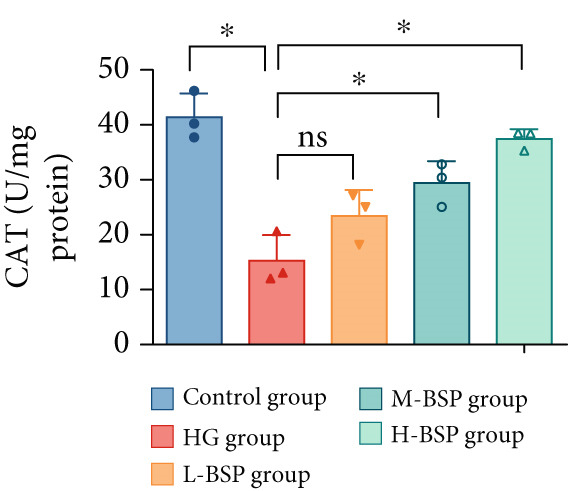
(d)

**Figure:**
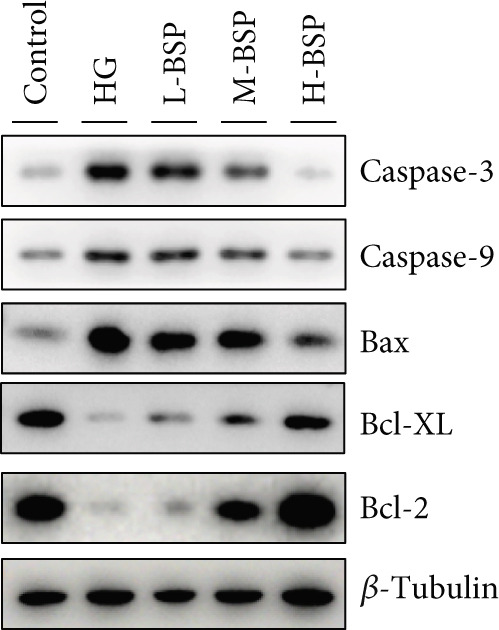
(e)

**Figure:**
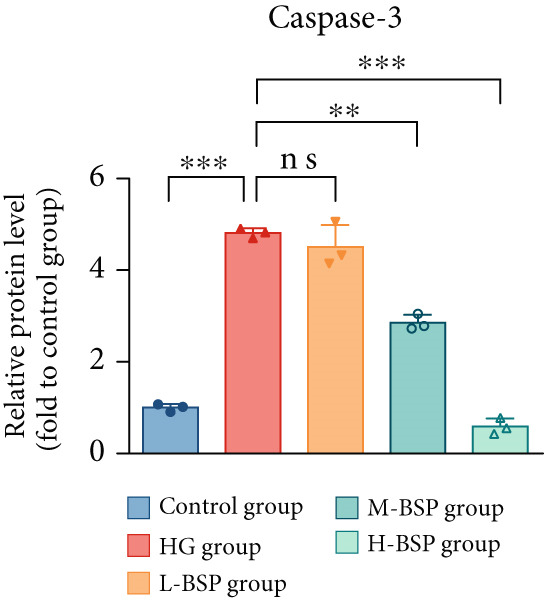
(f)

**Figure:**
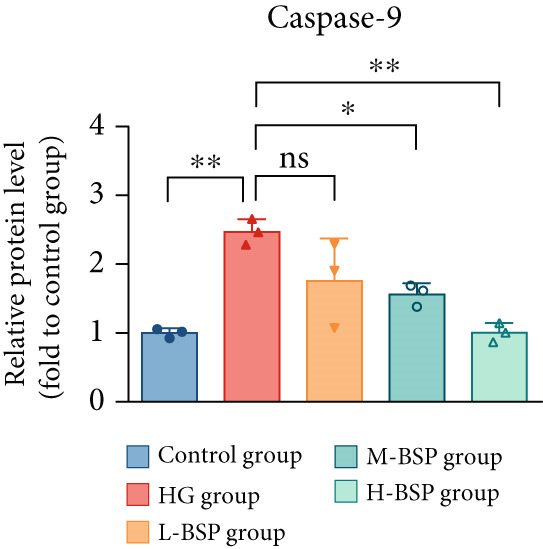
(g)

**Figure:**
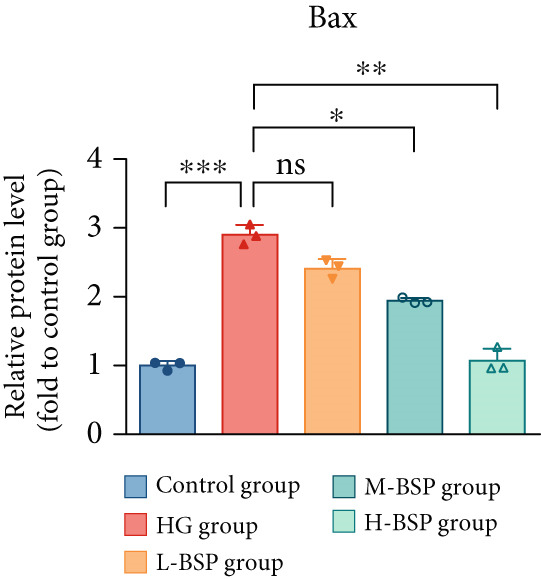
(h)

**Figure:**
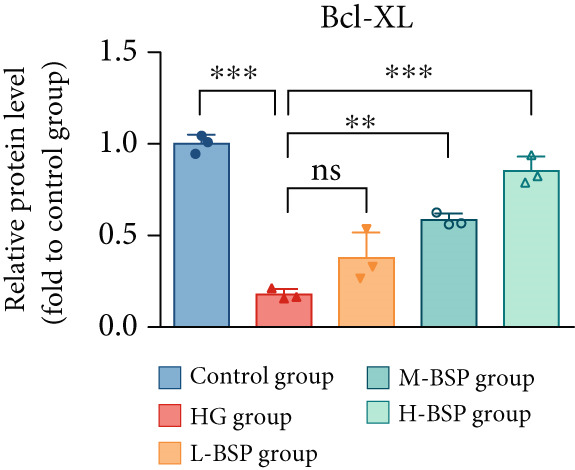
(i)

**Figure:**
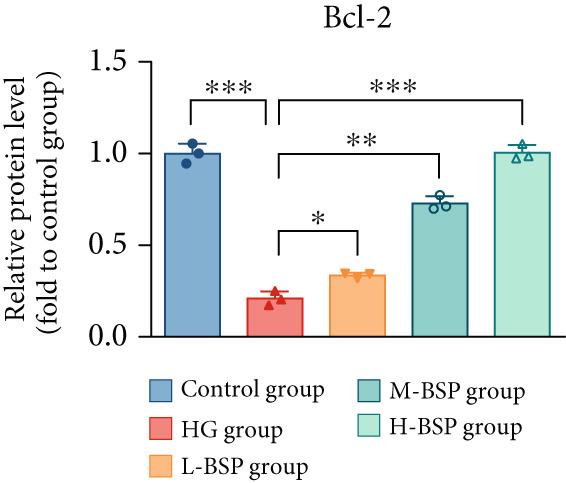
(j)

**Figure:**
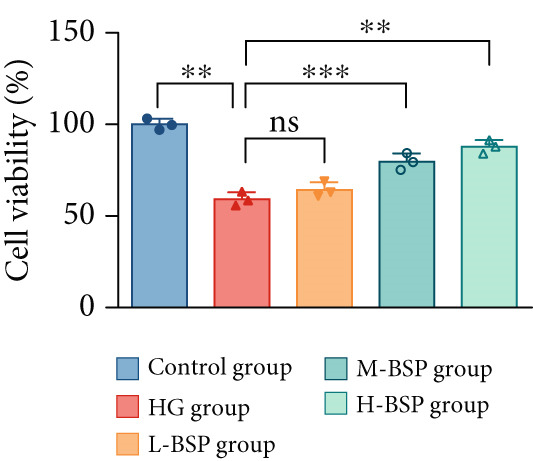
(k)

**Figure:**
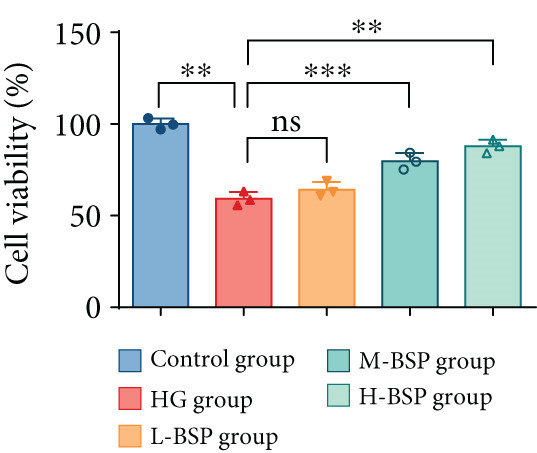
(l)

### 3.2. BSP Enhanced Tissue Regeneration in Diabetic Mice

Optimizing BSP′s dosage for treatment involved inducing skin defects on diabetic mice′s backs, with saline serving as a control. As shown in Figures 2b, 2c, and 2d, by Day 7, the wound area in the saline group had only reduced by about 33%, while the L‐BSP, M‐BSP, and H‐BSP groups experienced significant reductions of approximately 50%, 60%, and 71%, respectively. The wound areas in mice treated with H‐BSP were notably smaller by Day 14, contrasting with the saline group. High doses of BSP showed a positive influence on diabetic models.

Figure 2BSP promoted diabetic mice′s wound repair. (a) The process of establishing a diabetic mouse model and skin defect creation. (b) Images of diabetic wounds on Days 0, 3, 7, and 14 with BSP treatment at different concentrations. (c) Healing trace on Days 0, 3, 7, 10, and 14. (d) Relative wound area ratio at Days 3, 7, 10, and 14. (e) Masson′s trichrome staining on Day 14. Scale bar: 1 mm and 200 *μ*m. (f) H&E staining on Day 14. Scale bar: 1 mm and 200 *μ*m. HG: high glucose, BSP: *Bletilla striata* polysaccharide, STZ: streptozocin, H&E: hematoxylin and eosin. Data are expressed as the mean ± SD (*n* = 6).  ^∗^ *p* < 0.05,  ^∗∗^ *p* < 0.01, and  ^∗∗∗^ *p* < 0.001 versus the indicated groups. ns, no significant difference.

**Figure:**
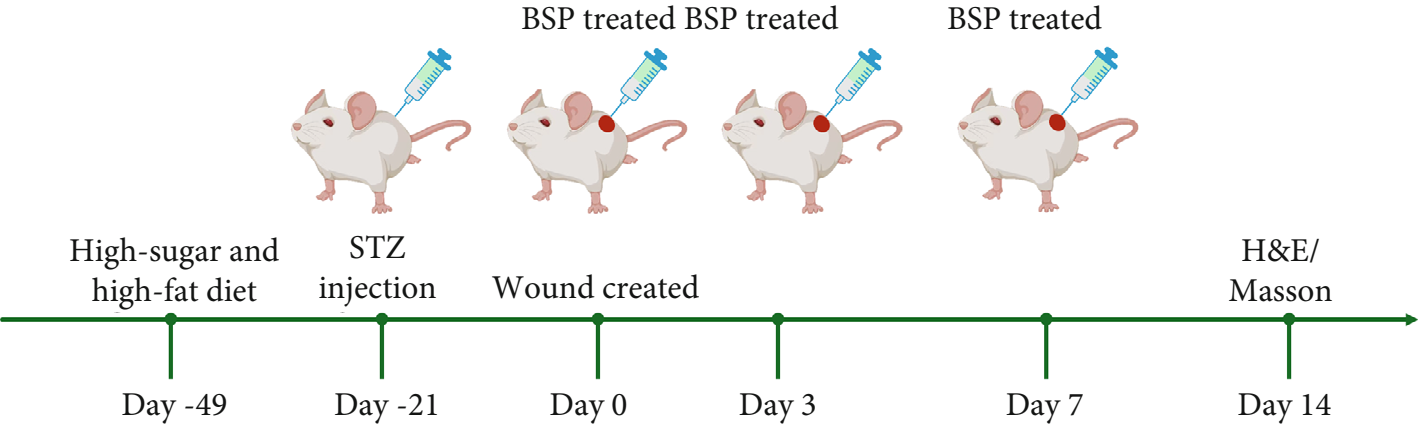
(a)

**Figure:**
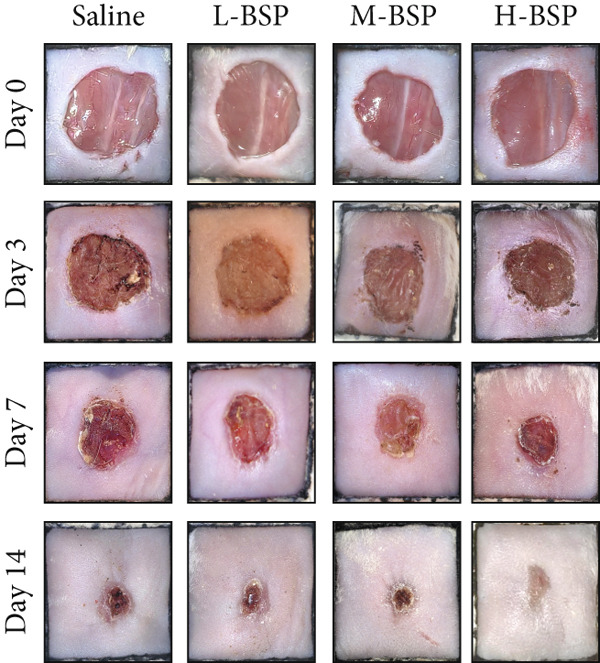
(b)

**Figure:**
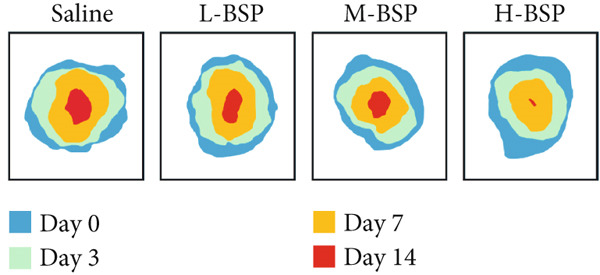
(c)

**Figure:**
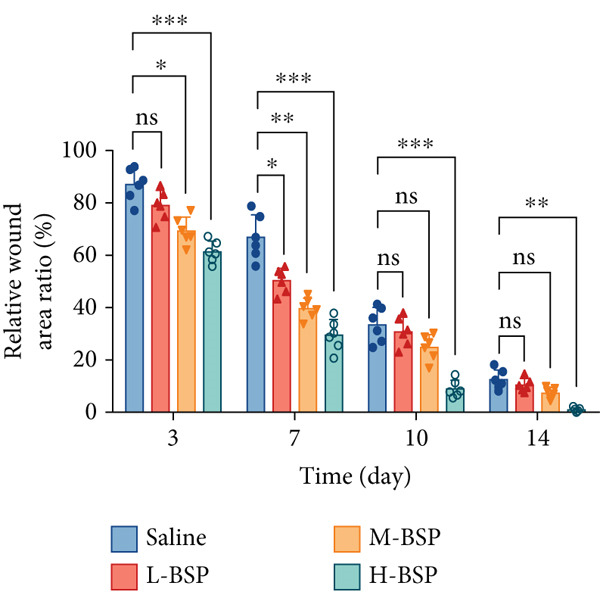
(d)

**Figure:**
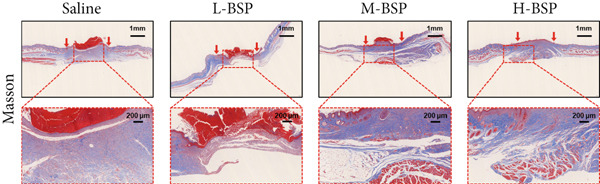
(e)

**Figure:**
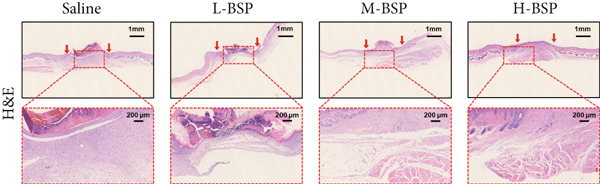
(f)

A panoramic view of the wounds, evaluated with Masson trichrome staining and H&E staining in the end, was presented in Figure 2e,f. We observed that the size of the wound scab decreased with increasing BSP concentration across all groups. Notably, collagen fiber deposition significantly increased, and inflammatory cells notably decreased in all groups when compared to the control. In particular, the H‐BSP group showed near‐complete wound re‐epithelialization, restored the hair follicle growth, and nearly total wound closure. Additionally, WB analysis of the wound tissues revealed that, compared to the control, mice injected with H‐BSP showed noteworthy downregulated Caspase‐3, Caspase‐9, and Bax. The expression of Bcl‐XL and Bcl‐2 was significantly upregulated, conversely. In contrast to the control, higher pretreatment BSP concentrations significantly elevated p‐PI3K and p‐Akt levels, along with nuclear Nrf2, while concurrently reducing cytoplasmic Nrf2 expression (Figures S4 and S5). In summary, these results collectively demonstrate that BSP treatment significantly accelerates diabetic wound healing in a concentration‐dependent manner, as evidenced by reduced wound size, enhanced collagen deposition, decreased inflammation, and favorable alterations in key protein expression and signaling pathways. Specifically, the downregulation of proapoptotic markers and upregulation of antiapoptotic markers in the H‐BSP group suggest a reduction in cell apoptosis, which may contribute to improved tissue repair and wound closure.

### 3.3. PI3K/Akt/Nrf2 Inhibitors Block the Antioxidant and Antiapoptosis Effects of BSP on L929 Cells

Three distinct inhibitors were introduced that target the PI3K/Akt/Nrf2 signaling cascade. As shown in Figures 3a, 3b, 3c, 3d, 3e, 3f, 3g, 3h, 3i, 3j, 3k, and 3l, oxidation system homeostasis was rescued after the intervention of BSP. Meanwhile, the result showed the improvement of antiapoptosis ability in L929 cells when pretreated with BSP under HG microenvironment.

Figure 3PI3K/Akt/Nrf2 inhibitors block the effects of BSP on L929 cells. (a–d) Level of MDA, GSH, SOD, and CAT of L929 cells. (e) WB images. (f–k) Quantitative analysis of WB. (l) Cell viability of L929 cells. HG: high glucose, BSP: *Bletilla striata* polysaccharide, MDA: malondialdehyde, GSH: glutathione, SOD: superoxide dismutase, CAT: catalase. Data are expressed as the mean ± SD (*n* = 3).  ^∗^ *p* < 0.05 and  ^∗∗^ *p* < 0.01 versus the indicated groups.

**Figure:**
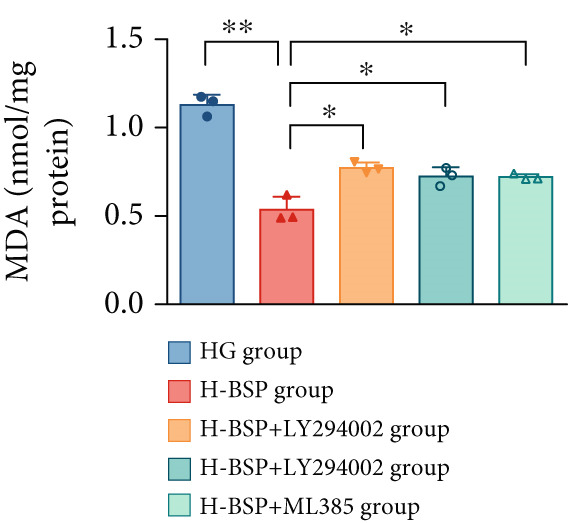
(a)

**Figure:**
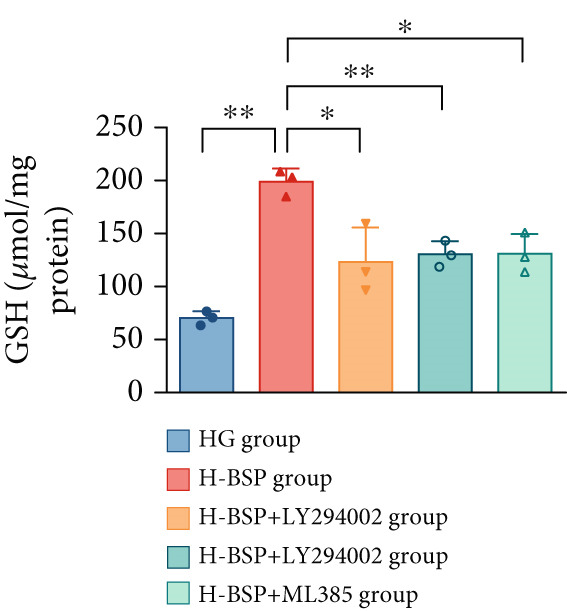
(b)

**Figure:**
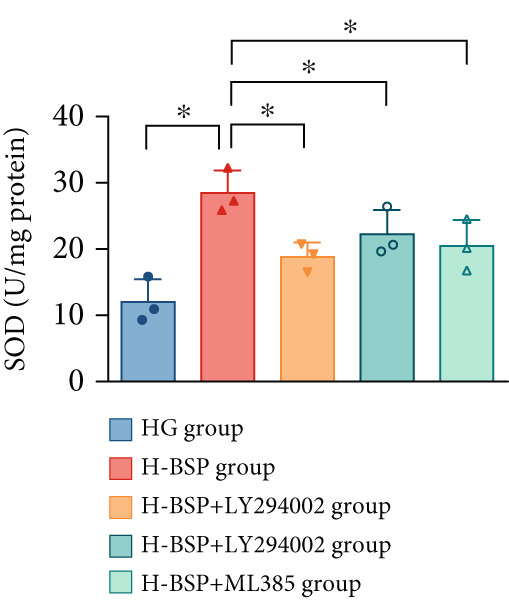
(c)

**Figure:**
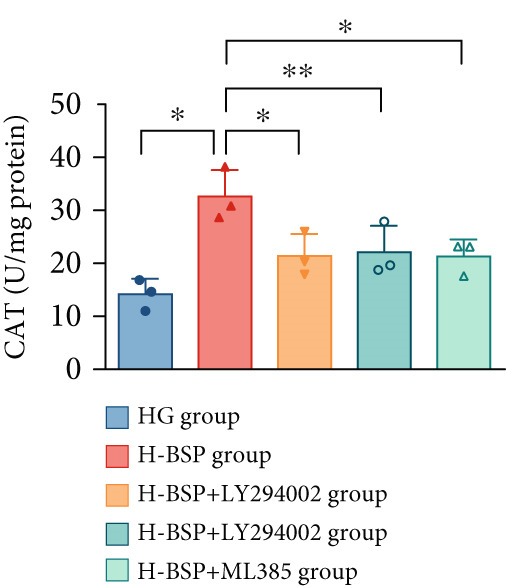
(d)

**Figure:**
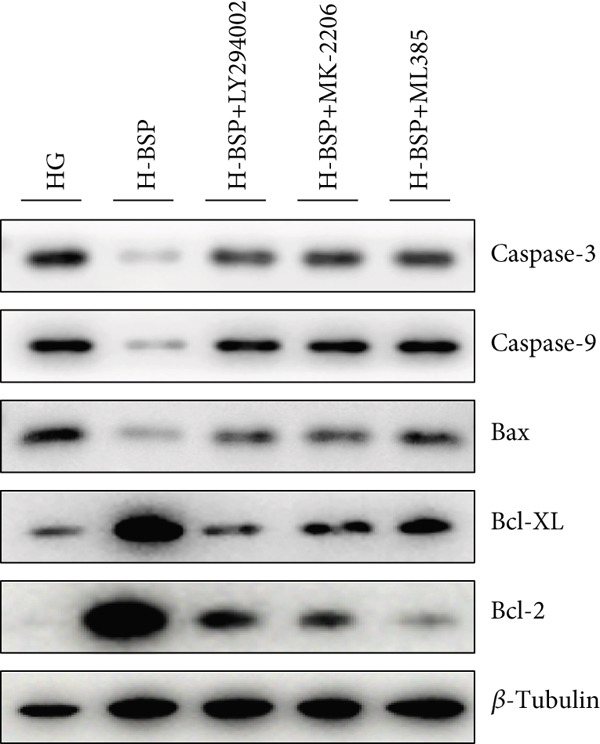
(e)

**Figure:**
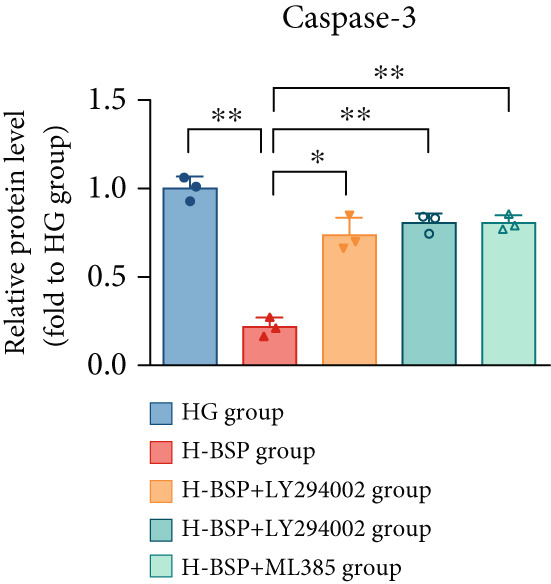
(f)

**Figure:**
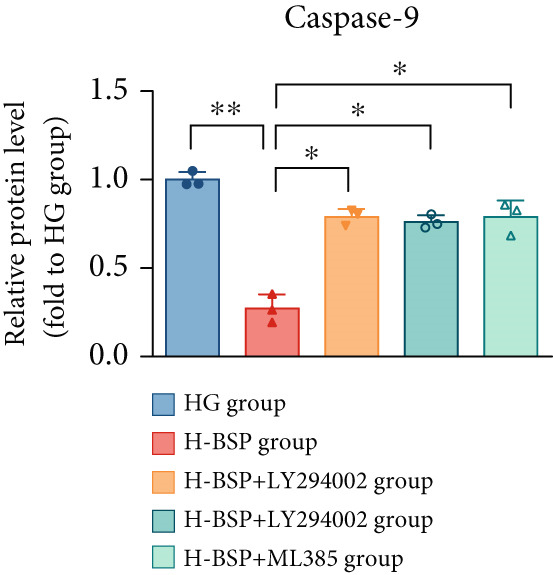
(g)

**Figure:**
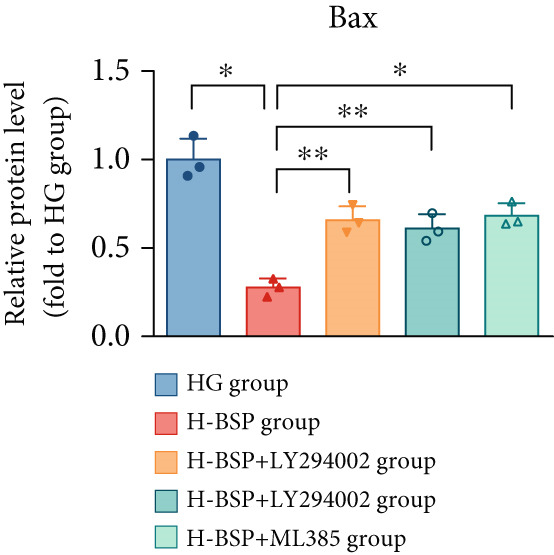
(h)

**Figure:**
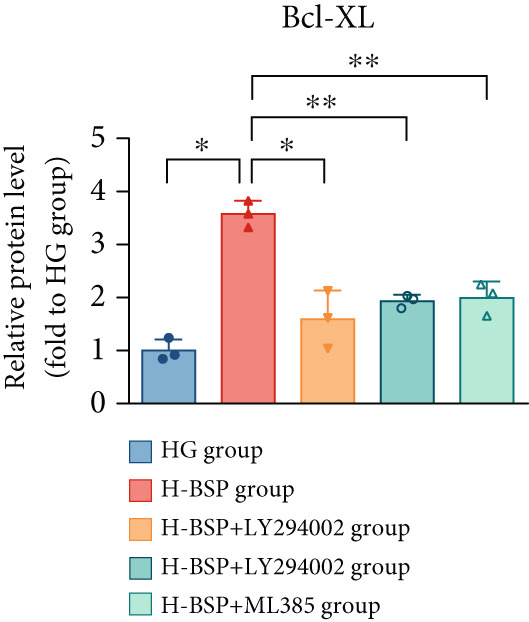
(i)

**Figure:**
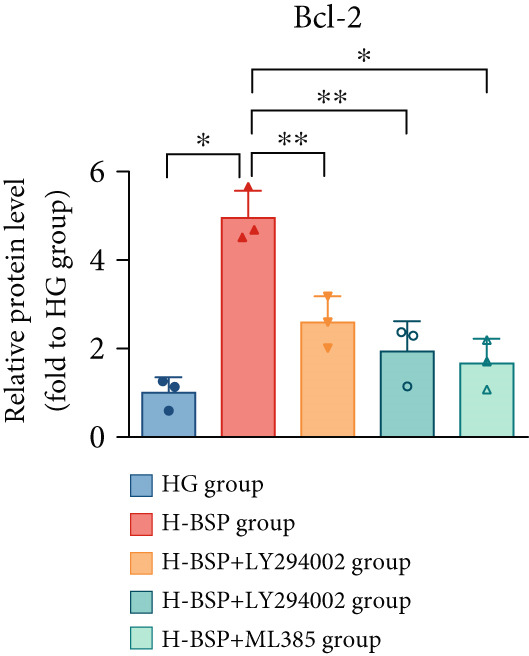
(j)

**Figure:**
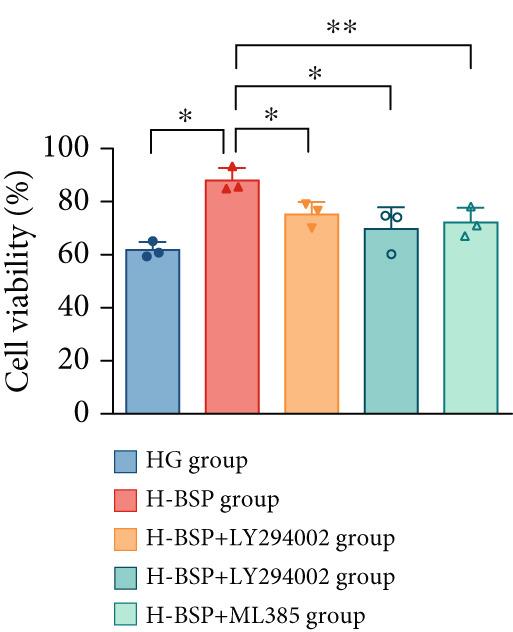
(k)

**Figure:**
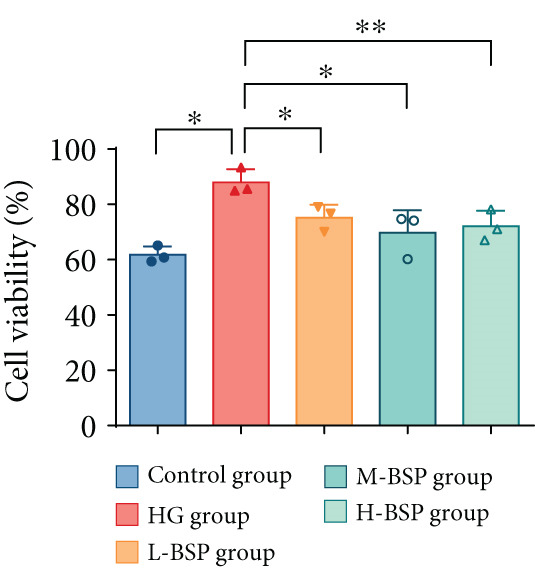
(l)

The PI3K inhibitor LY294002 has the capacity to inhibit the phosphorylation of PI3K. Compared with the H‐BSP group, the MDA level of the H‐BSP + LY294002 group significantly increased, and levels of GSH, SOD, and CAT significantly decreased. In contrast to the H‐BSP group, the protein expression levels of Caspase‐3, Caspase‐9, and Bax were significantly elevated, while those of Bcl‐XL and Bcl‐2 remarkably declined in the H‐BSP + LY294002 group, indicating the blockade of the PI3K/Akt/Nrf2 signaling pathway reverses the function of BSP on L292 cells. Additionally, cell viability markedly decreased due to the administration of LY294002. The ability of BSP to ameliorate apoptosis secondary to oxidative damage induced by a HG microenvironment in fibroblasts is possibly attributed to the phosphorylation of PI3K.

MK‐2206, as an inhibitor of Akt, has the effect of inhibiting Akt phosphorylation. In the H‐BSP + MK‐2206 group, the results showed the same trend as that in the H‐BSP + LY294002 group. These findings indicated that Akt phosphorylation is associated with the suppression of apoptosis secondary to oxidative damage induced by H‐BSP in HG‐treated L929 cells.

ML385 binds to Nrf2, altering the DNA affinity of the Nrf2‐MAFG complex. Upon comparing the H‐BSP + ML385 group with the H‐BSP group, it was observed that a similar outcome occurred in the H‐BSP + LY294002 group and H‐BSP + MK‐2206 group. In summary, BSP boosts the antioxidant capacity of the cells in the HG microenvironment through stimulating PI3K/Akt phosphorylation, which subsequently drives the nuclear translocation of Nrf2. Ultimately, this progress ameliorates the apoptosis‐related phenotype and promotes cell viability.

### 3.4. PI3K/Akt/Nrf2 Inhibitors Restrained BSP From Accelerating Diabetic Wound Healing

To elucidate the mechanisms underlying BSP‐induced diabetic wound healing, PI3K/Akt/Nrf2 inhibitors were introduced in vivo. As shown in Figures 4b, 4c, and 4d, on Day 3, the wound areas in the H‐BSP group exhibited a significant reduction compared to those in the saline group. Meanwhile, the healing speed was noticeably slower in the H‐BSP + LY294002, H‐BSP + MK‐2206, and H‐BSP + ML385 groups compared to the H‐BSP group. By Day 7, the wound areas in the H‐BSP group significantly decreased compared to the saline group, while the relative wound area ratios in the H‐BSP + LY294002, H‐BSP + MK‐2206, and H‐BSP + ML385 groups remained larger when compared to the H‐BSP group. On Day 14, the wounds in the H‐BSP group almost completely healed. The differences in relative wound area ratios between the H‐BSP + LY294002, H‐BSP + MK‐2206, and H‐BSP + ML385 groups and the H‐BSP group remained significant. This further suggests that BSP promotes wound healing through the PI3K/Akt/Nrf2 signaling axis.

Figure 4PI3K/Akt/Nrf2 inhibitors reduced the therapeutic effect of BSP on the diabetic mice. (a) The process of establishing a diabetic mouse model and skin defect creation. (b) Images of diabetic wounds on Days 0, 3, 7, and 14 with BSP treatment at different concentrations. (c) Healing trace on Days 0, 3, 7, 10, and 14. (d) Relative wound area ratio at Days 3, 7, 10, and 14. (e) Masson′s trichrome staining on Day 14. Scale bar: 1 mm and 200 *μ*m. (f) H&E staining on Day 14. Scale bar:1 mm and 200 *μ*m. BSP: *Bletilla striata* polysaccharide, STZ: streptozocin, H&E: hematoxylin and eosin. Data are expressed as the mean ± SD (*n* = 6).  ^∗^ *p* < 0.05,  ^∗∗^ *p* < 0.01, and  ^∗∗∗^ *p* < 0.001.

**Figure:**
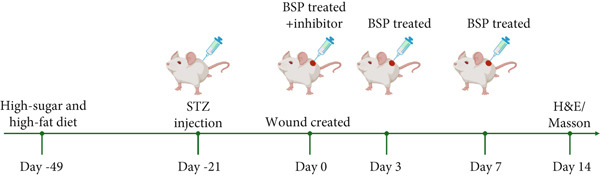
(a)

**Figure:**
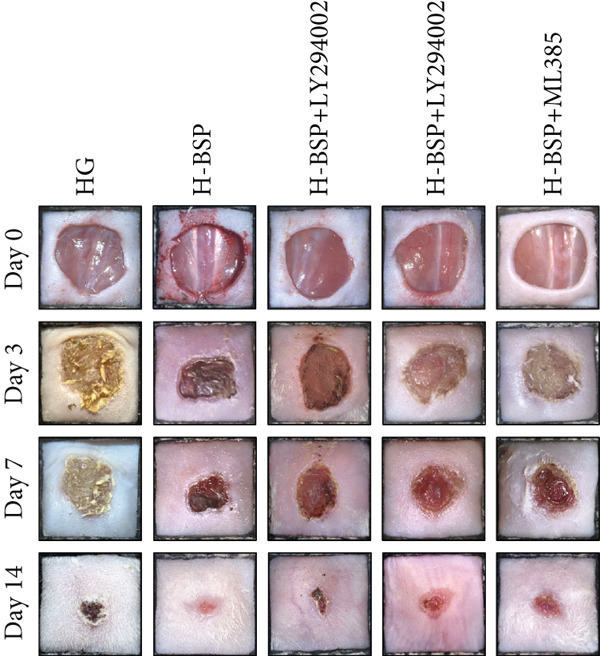
(b)

**Figure:**
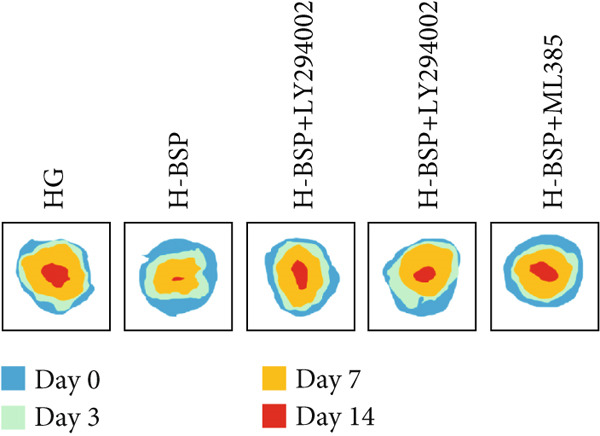
(c)

**Figure:**
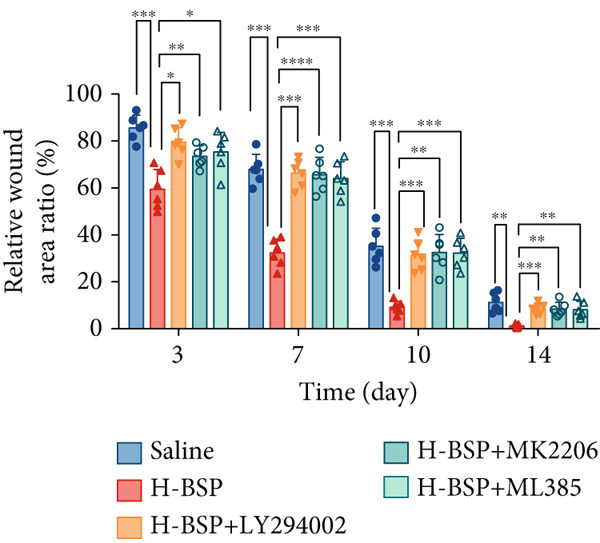
(d)

**Figure:**
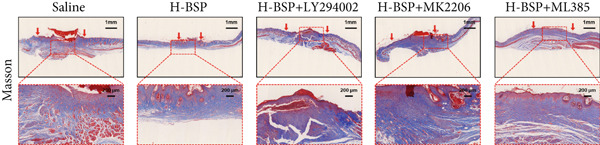
(e)

**Figure:**
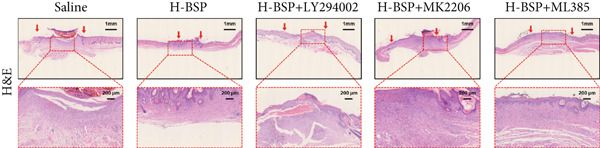
(f)

A comprehensive evaluation of the wounds via Masson trichrome staining and H&E staining on Day 14 is depicted in Figure 4e,f. We found that the H‐BSP group exhibited significantly enhanced wound healing compared to the saline group, as evidenced by robust collagen deposition, extensive re‐epithelialization, restored hair follicle growth, and diminished inflammatory symptoms. In contrast, the wound healing rates in the H‐BSP + LY294002, H‐BSP + MK‐2206, and H‐BSP + ML385 groups were much lower than those in the H‐BSP group. These groups exhibited relatively poor hair follicle growth, more pronounced inflammation, and overall, less effective wound healing than the H‐BSP group. The consistent experimental results demonstrate the mechanism of BSP in treating diabetic wounds.

### 3.5. BSP‐Mediated Antiapoptotic Effects in Diabetic Wounds Are Dependent on the PI3K/Akt/Nrf2 Pathway

The changes of diabetic wound samples from mice also demonstrated the effect of PI3K/Akt/Nrf2 signaling pathway activated by BSP on wound healing. The data presented in Figures 5a, 5b, 5c, 5d, 5e, and 5f clearly demonstrate that the H‐BSP treatment led to a notable decrease in the expression levels of Caspase‐3, Caspase‐9, and Bax proteins. Conversely, this same protocol caused a marked increase in Bcl‐XL and Bcl‐2 protein expression when compared against the saline group. Collectively, H‐BSP attenuates apoptosis in diabetic wound tissue cells by shifting the balance of Bcl‐2 family proteins and caspase activity toward an antiapoptotic state. H‐BSP + LY294002, H‐BSP + MK‐2206, and H‐BSP + ML385 groups all showed significant increases in Caspase‐3, Caspase‐9, and Bax expression, along with significant decreases in Bcl‐XL and Bcl‐2 levels compared to the H‐BSP group alone. The application of PI3K/Akt/Nrf2 signaling pathway inhibitors weakens the effects of H‐BSP. These findings demonstrate that BSP relieves oxidative damage, improves the antiapoptotic capacity of wound cells, and promotes the healing of diabetic wounds in vivo via the PI3K/Akt/Nrf2 signaling pathway. The results in experiments of diabetic models were consistent with those of cell experiments.

Figure 5BSP and PI3K/Akt/Nrf2 pathway inhibitors regulate the expression of apoptosis‐related proteins in wound tissues of diabetic mice. (a) WB images. (b–f) Quantitative analysis of WB. BSP: *Bletilla striata* polysaccharide, STZ: streptozocin, H&E: hematoxylin and eosin. Data are expressed as the mean ± SD (*n* = 3).  ^∗^ *p* < 0.05 and  ^∗∗^ *p* < 0.01 versus the indicated groups.

**Figure:**
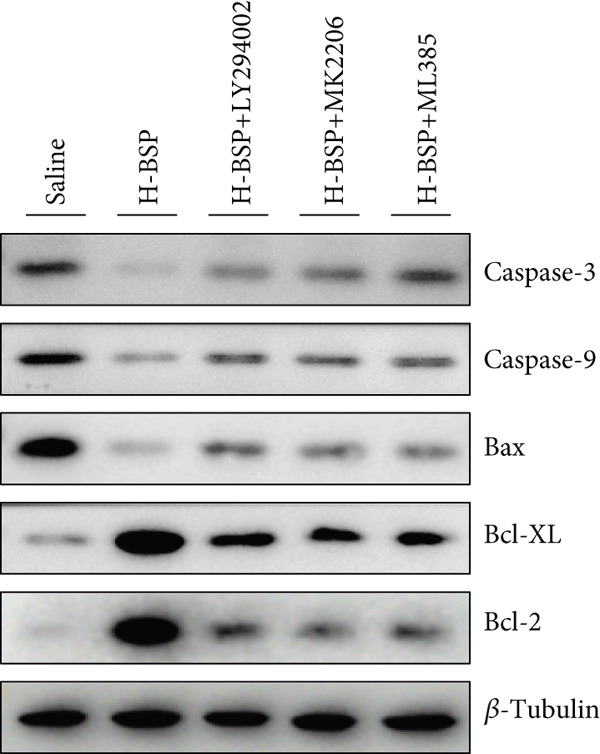
(a)

**Figure:**
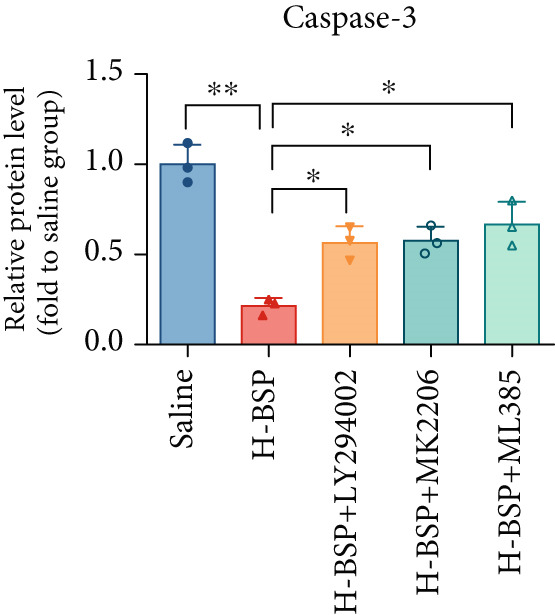
(b)

**Figure:**
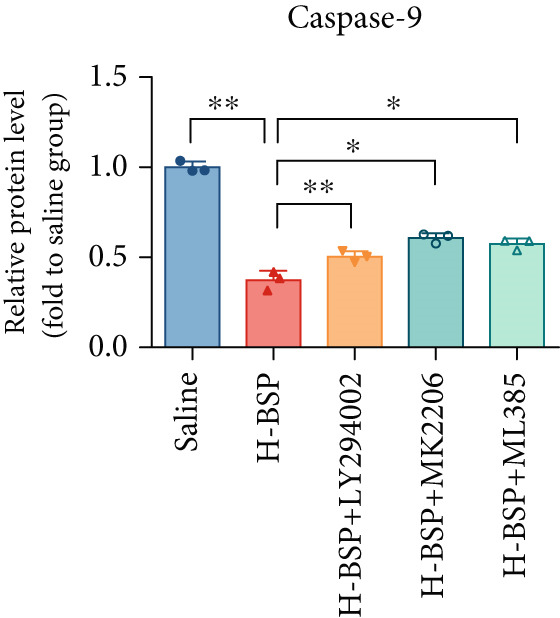
(c)

**Figure:**
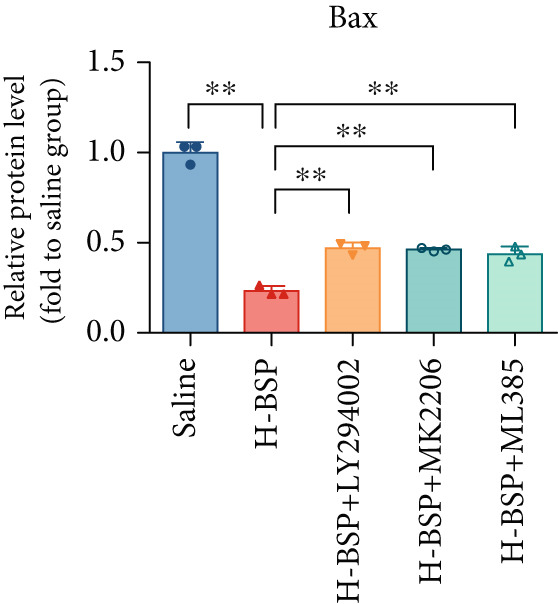
(d)

**Figure:**
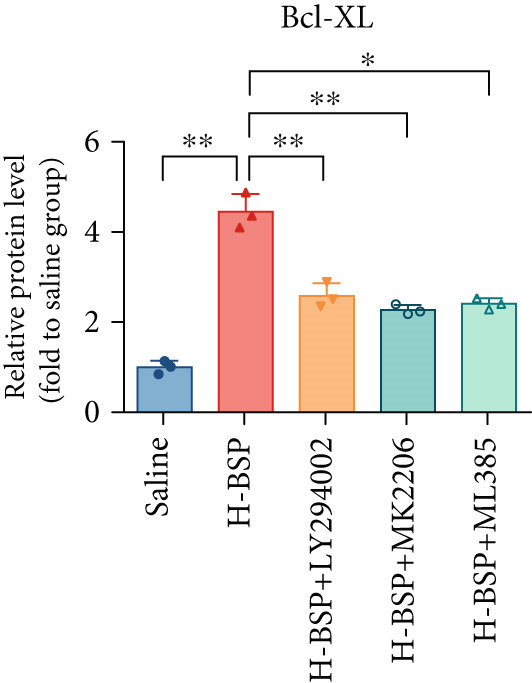
(e)

**Figure:**
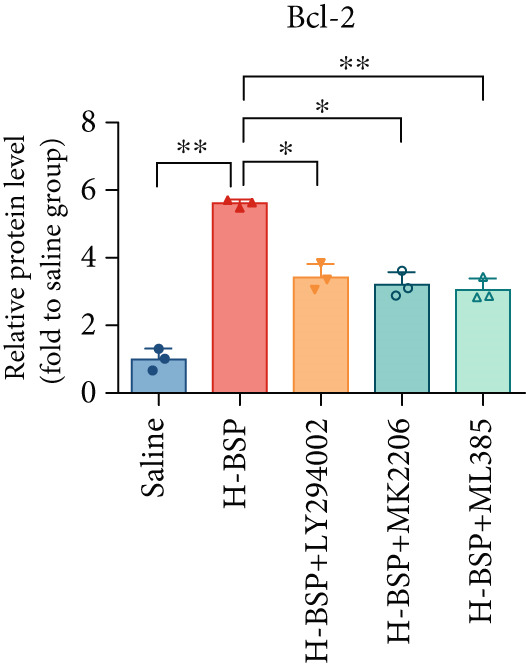
(f)

## 4. Discussion

Diabetic wounds pose a major clinical problem due to their complex pathophysiology characterized by impaired angiogenesis, chronic inflammation, and OS, which collectively impede the normal wound healing process [21]. Elevated ROS levels in diabetic wounds are primarily driven by the hyperglycemic microenvironment, which induces OS and subsequent cellular dysfunction, including apoptosis [22]. This not only damages cellular components but also inhibits collagen synthesis, thereby delaying wound closure and increasing the risk of infection [23]. Therefore, targeting OS and its downstream effects, such as apoptosis, is a promising strategy for improving diabetic wound healing. L929 murine fibroblasts are widely employed for wound healing and OS studies because of their high reproducibility and transfection compatibility; therefore, they are selected for the initial mechanistic screening of antioxidant/antiapoptotic signaling of BSP. In our study, we found that BSP possesses excellent antioxidant function, which could prevent L929 cells from apoptosis in HG conditions. Moreover, BSP intervention significantly accelerated the repair of diabetic wounds.

OS results from an imbalance between ROS production and the body′s capacity for detoxification or repair [24]. MDA is a widely used biomarker of lipid peroxidation and oxidative damage, which are highly related to diabetic retinopathy [25] and nephropathy [26]. Elevated levels of MDA indicate high OS, disrupting the balance of pro‐ and anti‐inflammatory mediators which finally leads to impaired wound healing in diabetic patients [27]. In contrast, SOD, GSH, and CAT are essential antioxidant enzymes. SOD transforms superoxide radicals into oxygen and hydrogen peroxide. GSH acts as a major intracellular antioxidant and detoxifying agent. In addition, CAT decomposes hydrogen peroxide to water and oxygen. In diabetic wounds, the activities of these antioxidant enzymes are often suppressed, leading to a further increase in OS, prolonged inflammation, and tissue damage [28, 29]. A glucose gradient experiment was set, and 30 mM glucose medium induced the most significant OS in L292 cells, decreasing the cell viability dramatically. However, BSP pretreatment significantly reduced MDA levels while enhancing the activities of SOD, GSH, CAT, and cell viability. This effect appears to be dose‐dependent and reaches the peak at a concentration of 1 mg/mL.

Natural polysaccharide has shown its potential for DM treatment in recent years. Okra polysaccharide (OP) has been proven to decrease blood glucose and insulin sensitivity in Type 2 diabetic mice. Concurrently, OP treatment has been shown to enhance the endogenous antioxidant defense system within hepatic tissue. The molecular mechanisms underpinning these beneficial effects appear to involve the strategic activation of the PI3K/Akt/GSK3*β* signaling cascade, a critical intracellular pathway that regulates glucose uptake and metabolism [30]. BSP, characterized by its solubility and noncellulosic nature, exhibits a wide array of biological activities that have garnered significant interest in both academic and clinical circles. BSP′s multifunctional properties include hemostatic effects, which are crucial for preventing excessive bleeding, antibacterial capabilities that offer protection against infectious pathogens, antitumor actions that may contribute to cancer therapy, antifibrotic functions that help mitigate the formation of excessive connective tissue, antioxidant effects that combat cellular aging, and low toxicity levels, which are essential for its safety and efficacy in therapeutic applications. Research in this area continues to explore the potential of BSP as a bioactive compound with wide‐ranging therapeutic applications, offering a glimpse into the vast potential of natural products in modern medicine [31].

Dysregulation of apoptosis, particularly in the context of diabetic wounds, contributes to impaired healing and chronic tissue damage [32]. In the intrinsic apoptotic process, proteins like Caspase‐9, Caspase‐3, Bax, Bcl‐2, and Bcl‐XL play crucial roles. This pathway is often sparked by cellular stressors such as OS, genetic harm, or a lack of growth factors [33]. Bax, Bcl‐XL, and Bcl‐2 are key controllers of mitochondrial stability. Bax functions as a proapoptotic factor, enhancing mitochondrial outer membrane permeabilization (MOMP), thus triggering Cytochrome c (Cyto‐C) discharge and initiating the caspase cascade activation. In contrast, Bcl‐2 and Bcl‐XL inhibit this process and maintain mitochondrial integrity, thereby protecting cells from apoptosis [34, 35]. After release from the chondriosome, Cyto‐C binds to apoptotic protease activating factor‐1 (Apaf‐1), creating the apoptosome to trigger Caspase‐9. Caspase‐9 serves as the linchpin in the apoptotic pathway, springing into action when Cyto‐C is freed from the mitochondrial prison. Once activated, this initiator caspase goes to work on downstream effector caspases, with Caspase‐3 taking center stage as the primary executioner that dismantles various cellular components. This domino effect ultimately triggers the hallmark features of programmed cell death—DNA breakdown, cellular shrinkage, and the formation of blister‐like membrane protrusions that signal the cell′s impending demise [36]. In our study, we analyze the expression of these proteins by WB after the administration of BSP to elucidate the effects of BSP on cells and wounds in HG environments. The experiments demonstrated the anticellular apoptosis ability of BSP, along with enhancing the wound healing process. Therefore, our findings highlight that BSP intervention is a promising therapeutic regimen for diabetic wounds.

The PI3K/Akt signaling axis is pivotal in regulating cell proliferation, senescence, programmed cell death, and metabolic processes [37]. The PI3K/Akt pathway could promote protein synthesis and cell proliferation by activating rapamycin (mTOR) and stabilizing *β*‐catenin by inhibiting GSK3, thereby promoting cell survival and migration [38]. Moreover, Akt can phosphorylate the proapoptotic protein Bad, rendering it inactive and preventing its binding to the antiapoptotic protein Bcl‐XL, which inhibits Bax from forming channels in the mitochondrial membrane, thus inhibiting Cyto‐C release and preventing apoptosis [39]. Research confirmed that early PI3K/Akt pathway activation reduces Caspase‐9 and Caspase‐3 levels, limiting apoptosis and inflammation in post–spinal cord trauma [40]. Akt could also phosphorylate forkhead box O (FOXO) family transcription factors, inhibiting their nuclear translocation and thereby diminishing production of apoptosis‐promoting genes including Bin, FasL, TRAIL, and Bax [41]. The Nrf2/ARE pathway is closely related to antioxidant gene transcription, including GSH, SOD1, NAD(P)H, NQO1, and HO‐1 [42]. However, under an HG environment, it was also inhibited, leading to disrupted intracellular redox homeostasis. Experimental investigations utilizing genetically modified murine models have demonstrated a pronounced impairment in the cutaneous wound healing process among Nrf2‐deficient (Nrf2^−/−^) subjects when compared to their wild‐type counterparts (Nrf2^+/+^) within the context of a diabetic condition. Using standardized wound measurement techniques such as thermal imaging and histological analysis, the wounds were found to heal more slowly in Nrf2^−/−^ mice. This delayed closure was characterized by increased oxidative DNA damage, apoptosis, and MMP‐9 levels, alongside reduced granulation tissue and TGF‐*β*1 expression. Together, these results indicate that Nrf2 deficiency worsens the wound healing impairment in diabetes [43].

**Figure 6:**
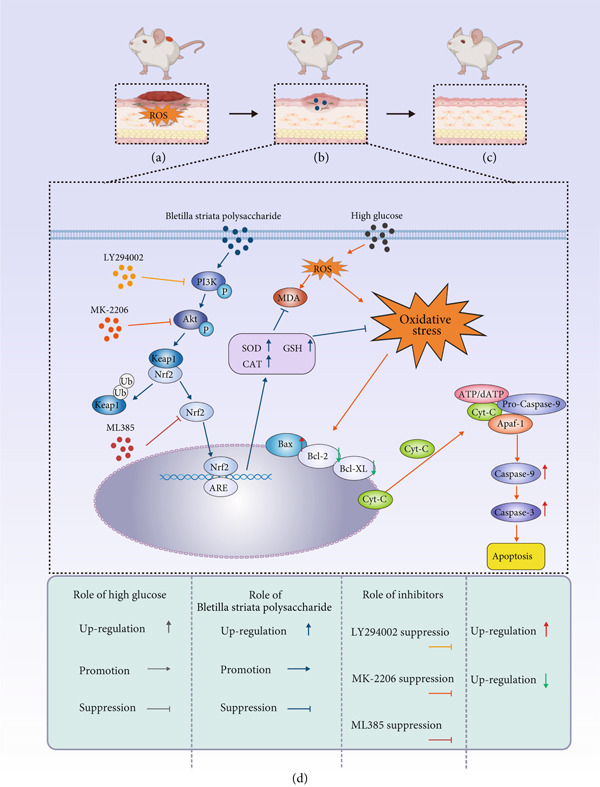
Mechanism of BSP in the treatment of diabetic wounds. (a) Schematic diagram of the diabetic wound mechanism in mice. (b, d) Mechanism of BSP on diabetic wounds in mice. (c) Diagram of the cured mice′s skin.

In our work, the intervention of 30 mM glucose on L292 cells decreased the protein expressions of p‐PI3K, p‐Akt, and nuclear Nrf2. However, this effect was reversed after the administration of varying concentrations of BSP. We hypothesize that BSP protects L292 cells from apoptosis and promotes diabetic wound healing via the PI3K/Akt/Nrf2 pathway. To verify our hypothesis, inhibitors for PI3K, Akt, and Nrf2 were used before BSP intervention. The findings demonstrate that BSP could promote the phosphorylation of PI3K and Akt, facilitating the nuclear translocation of Nrf2 under pathological conditions. However, the beneficial effects of BSP were suppressed after the introduction of LY294002, MK‐2206, and ML385 (Figures 3, 4, 5, and 6). The overall mechanism is demonstrated in Figure 6. Our findings indicate that BSP‐mediated activation of the PI3K/Akt/Nrf2 pathway may account for its ability to promote diabetic wound healing, revealing the potential of BSP for diabetic wound treatment as a natural medicine and paving the way for its wider clinical use.

There are some limitations of this article. First, the exclusive reliance on a single immortalized fibroblast line may overestimate the protective potency of BSP because L929 cells do not fully capture the metabolic memory, epigenetic alterations, or chronic low‐grade inflammation that characterize primary dermal fibroblasts from diabetic individuals. Future studies should compare BSP‐elicited responses in primary fibroblasts isolated from diabetic versus nondiabetic mice to enhance physiological relevance. Second, the excisional wound model was monitored for only 14 days, precluding conclusions about the quality of regenerated tissue, scar remodeling, or long‐term recurrence. Third, we fully acknowledge that the STZ‐induced diabetic model does not fully replicate the complex pathophysiology of human T2DM, particularly in terms of insulin resistance progression, metabolic inflammation, and *β*‐cell dysfunction kinetics. It should be noted that we employed a high‐fat diet + low‐dose STZ protocol, which is intended to partially replicate the dual defects of insulin resistance and *β*‐cell dysfunction seen in human T2DM, rather than the classic high‐dose STZ model that induces near‐total *β*‐cell ablation. Finally, pharmacokinetic profiles and toxicity in vivo of BSP have not yet been studied, all of which are prerequisites for clinical translation. Moving forward, BSP could be formulated into a stable, nontoxic, degradable, and injectable hydrogel and validated into a large animal diabetic wound model. Randomized controlled trials will then decide whether this natural product is ready for clinical use.

## 5. Conclusion

In conclusion, BSP is a significant option for inhibiting apoptosis in L929 cells to promote wound healing in the background of diabetic wound therapy. In short, BSP can increase the antioxidant capacity of L929 cells under a HG microenvironment by activating the PI3K/Akt/Nrf2 signaling pathway, thereby alleviating the oxidative damage of L929 cells, and then improving the apoptosis‐related phenotype of L929 cells to enhance the cell viability, thus improving collagen deposition and reducing inflammation in diabetic wounds and accelerating wound healing.

The present study provides a promising novel therapeutic idea for the treatment of diabetic wounds and provides relevant experimental and theoretical bases for related research.

## Conflicts of Interest

The authors declare no conflicts of interest.

## Author Contributions

Gang Xu: conceptualization, validation, resources, writing—review and editing, and supervision; Shuangyi Xu: methodology, resources, and visualization; Zerui Ni: formal analysis, investigation, data curation, writing—original draft, and visualization; Tong Zhang: formal analysis and investigation; Xiaowei Zhang: investigation and data curation; Xiaomei Li: resources and data curation; Limin Bai: resources and data curation; Lu Yu: data curation.

## Funding

This work was supported by Jiangsu Provincial Bureau of Traditional Chinese Medicine (YB2020086).

## Supporting information


**Supporting Information** Additional supporting information can be found online in the Supporting Information section. Figure S1 HG‐induced oxidative damage and apoptosis of L929 cells. (A–D) Level of MDA, GSH, SOD, and CAT of L929 cells under different treatments. (E) WB analysis of Caspase‐3, Caspase‐9, Bax, Bcl‐XL, and Bcl‐2 of L929 cells under different treatments. (F–K) Quantitative analysis of Caspase‐3, Caspase‐9, Bax, Bcl‐XL, and Bcl‐2 of L929 cells under different treatments. (L) Cell viability of L929 cells under different treatments. Figure S2: HG suppressed the phosphorylation of PI3K/Akt and accelerated Nrf2 nuclear translocation of L929 cells. (A, B) WB analysis of p‐PI3K, PI3K, p‐Akt, and Akt of L929 cells. (C, D) Quantitative analysis of p‐PI3K/PI3K and p‐Akt/Akt of L929 cells. (E, F) WB analysis of cytoplasmic and nuclear Nrf2 of L929 cells. (G, H) Quantitative analysis of cytoplasmic Nrf2 and nuclear Nrf2 of L929 cells. Figure S3: BSP improved the phosphorylation of PI3K/Akt and accelerated Nrf2 nuclear translocation in L929 cells. (A, B) WB analysis of p‐PI3K, PI3K, p‐Akt, and Akt of L929 cells. (C, D) Quantitative analysis of p‐PI3K/PI3K and p‐Akt/Akt of L929 cells. (E, F) WB analysis of cytoplasmic and nuclear Nrf2 of L929 cells. (G, H) Quantitative analysis of cytoplasmic Nrf2 and nuclear Nrf2 of L929 cells. Figure S4: BSP alleviated the apoptosis of L929 cells to accelerate diabetic wound healing in vivo. (A) WB analysis of Caspase‐3, Caspase‐9, Bax, Bcl‐XL, and Bcl‐2 of diabetic wound tissue. (B–F) Quantitative analysis of Caspase‐3, Caspase‐9, Bax, Bcl‐XL, and Bcl‐2 of diabetic wound tissue. Figure S5: BSP improved the phosphorylation of PI3K/Akt and accelerated Nrf2 nuclear translocation in vivo. (A, B) WB analysis of p‐PI3K, PI3K, p‐Akt, and Akt of diabetic wound tissue. (C, D) Quantitative analysis of p‐PI3K/PI3K and p‐Akt/Akt of diabetic wound tissue. (E, F) WB analysis of cytoplasmic and nuclear Nrf2 of diabetic wound tissue. (G, H) Quantitative analysis of cytoplasmic Nrf2 and nuclear Nrf2 of diabetic wound tissue.

## Data Availability

The data that support the findings of this study are available from the corresponding authors upon reasonable request.
