# Supplementary material for: Targeting Oxidative Stress and Apoptosis via PI3K/Akt/Nrf2 Pathway: The Therapeutic Role of Bletilla striata Polysaccharide in Diabetic Wound Repair
Source: J Diabetes Res. 2026 Jan 28;2026:5751331. doi: 10.1155/jdr/5751331 (PMC12851404; doi:10.1155/jdr/5751331)
Supplement: Supplementary file 1 — Supporting Information Additional supporting information can be found online in the Supporting Information section. Figure S1 HG‐induced oxidative damage and apoptosis of L929 cells. (A–D) Level of MDA, GSH, SOD, and CAT of L929 cells under different treatments. (E) WB analysis of Caspase‐3, Caspase‐9, Bax, Bcl‐XL, and Bcl‐2 of L929 cells under different treatments. (F–K) Quantitative analysis of Caspase‐3, Caspase‐9, Bax, Bcl‐XL, and Bcl‐2 of L929 cells under different treatments. (L) Cell viability of L929 cells under different treatments. Figure S2: HG suppressed the phosphorylation of PI3K/Akt and accelerated Nrf2 nuclear translocation of L929 cells. (A, B) WB analysis of p‐PI3K, PI3K, p‐Akt, and Akt of L929 cells. (C, D) Quantitative analysis of p‐PI3K/PI3K and p‐Akt/Akt of L929 cells. (E, F) WB analysis of cytoplasmic and nuclear Nrf2 of L929 cells. (G, H) Quantitative analysis of cytoplasmic Nrf2 and nuclear Nrf2 of L929 cells. Figure S3: BSP improved the phosphorylation of PI3K/Akt and accelerated Nrf2 nuclear translocation in L929 cells. (A, B) WB analysis of p‐PI3K, PI3K, p‐Akt, and Akt of L929 cells. (C, D) Quantitative analysis of p‐PI3K/PI3K and p‐Akt/Akt of L929 cells. (E, F) WB analysis of cytoplasmic and nuclear Nrf2 of L929 cells. (G, H) Quantitative analysis of cytoplasmic Nrf2 and nuclear Nrf2 of L929 cells. Figure S4: BSP alleviated the apoptosis of L929 cells to accelerate diabetic wound healing in vivo. (A) WB analysis of Caspase‐3, Caspase‐9, Bax, Bcl‐XL, and Bcl‐2 of diabetic wound tissue. (B–F) Quantitative analysis of Caspase‐3, Caspase‐9, Bax, Bcl‐XL, and Bcl‐2 of diabetic wound tissue. Figure S5: BSP improved the phosphorylation of PI3K/Akt and accelerated Nrf2 nuclear translocation in vivo. (A, B) WB analysis of p‐PI3K, PI3K, p‐Akt, and Akt of diabetic wound tissue. (C, D) Quantitative analysis of p‐PI3K/PI3K and p‐Akt/Akt of diabetic wound tissue. (E, F) WB analysis of cytoplasmic and nuclear Nrf2 of diabetic wound tissue. (G, H) Quanti [file JDR-2026-5751331-s001.docx]

Supplementary data


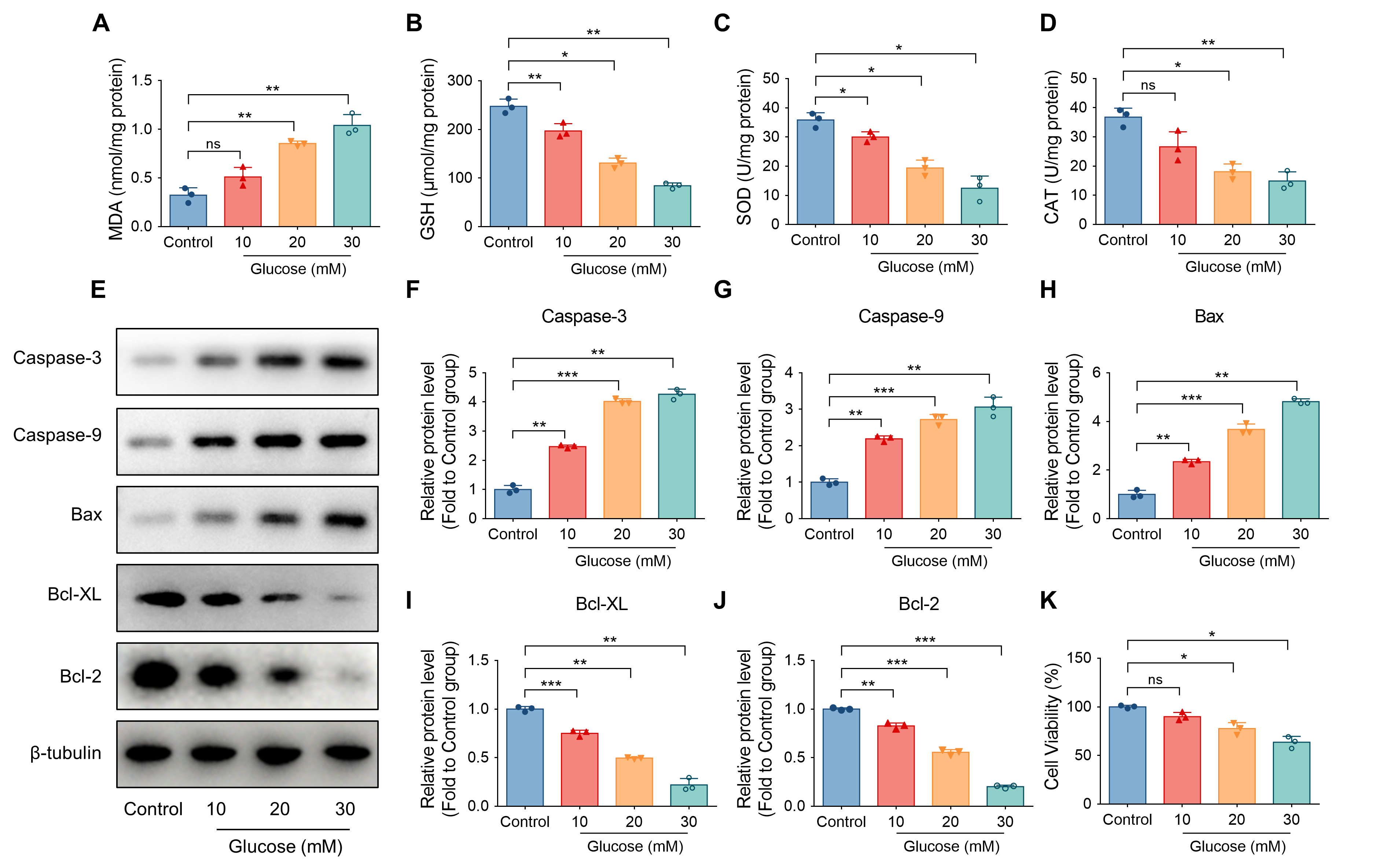


**Figure S1.** **HG induced oxidative damage and apoptosis of L929 cells.** (A-D) Level of MDA, GSH, SOD, and CAT of L929 cells under different treatment. (E) WB analysis of Caspase-3, Caspase-9, Bax, Bcl-XL and Bcl-2 of L929 cells under different treatment. (F-K) Quantitative analysis of Caspase-3, Caspase-9, Bax, Bcl-XL and Bcl-2 of L929 cells under different treatment. (L) Cell viability of L929 cells under different treatment. HG: high glucose, MDA: malondialdehyde, GSH: glutathione, SOD：superoxide dismutase, CAT: catalase. Data are expressed as the mean ± SD (n = 3).**P*<0.05, ***P*<0.01, ****P*<0.001, vs the indicated groups. NS, no significant difference.


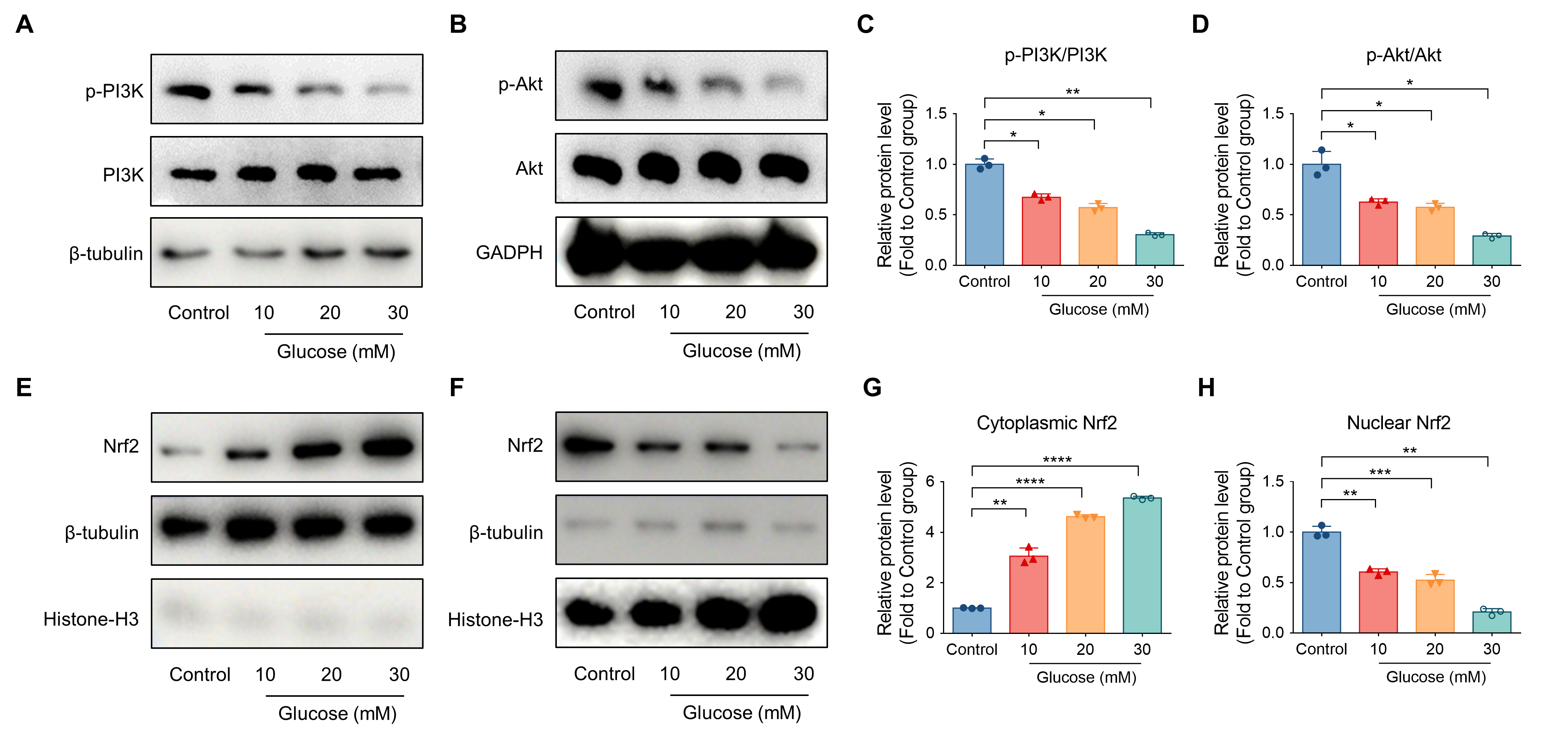


**Figure S2. HG suppressed the phosphorylation of PI3K/Akt and accelerated Nrf2 nuclear translocation of L929 cells.** (A and B) WB analysis of p-PI3K, PI3K, p-Akt, and Akt of L929 cells. (C and D) Quantitative analysis of p-PI3K/ PI3K and p-Akt/Akt of L929 cells. (E and F) WB analysis of cytoplasmic and nuclear Nrf2 of L929 cells. (G and H) Quantitative analysis of cytoplasmic Nrf2 and nuclear Nrf2 of L929 cells. Data are expressed as the mean ± SD (n = 3). **P<*0.05*, **P<*0.01, ****P*<0.001, vs the indicated groups. NS, no significant difference.


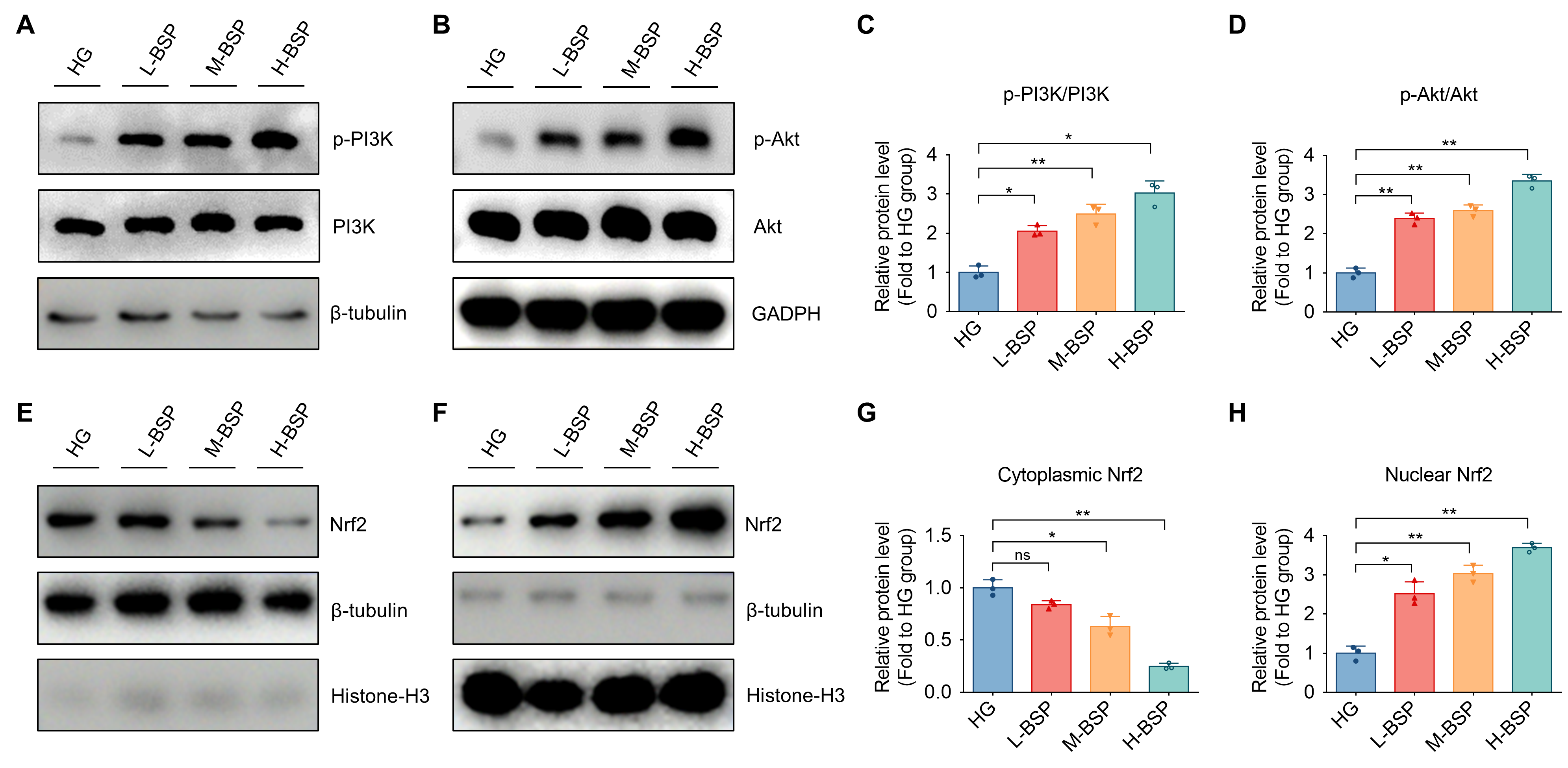


**Figure S3. BSP improved the phosphorylation of PI3K/Akt and accelerated Nrf2 nuclear translocation L929 cells.** (A and B) WB analysis of p-PI3K, PI3K, p-Akt, and Akt of L929 cells. (C and D) Quantitative analysis of p-PI3K/ PI3K and p-Akt/Akt of L929 cells. (E and F) WB analysis of cytoplasmic and nuclear Nrf2 of L929 cells. (G and H) Quantitative analysis of cytoplasmic Nrf2 and nuclear Nrf2 of L929 cells. HG: high glucose, BSP: bletilla striata polysaccharide. Data are expressed as the mean ± SD (n = 3). **P<*0.05*, **P<*0.01, vs the indicated groups. NS, no significant difference.


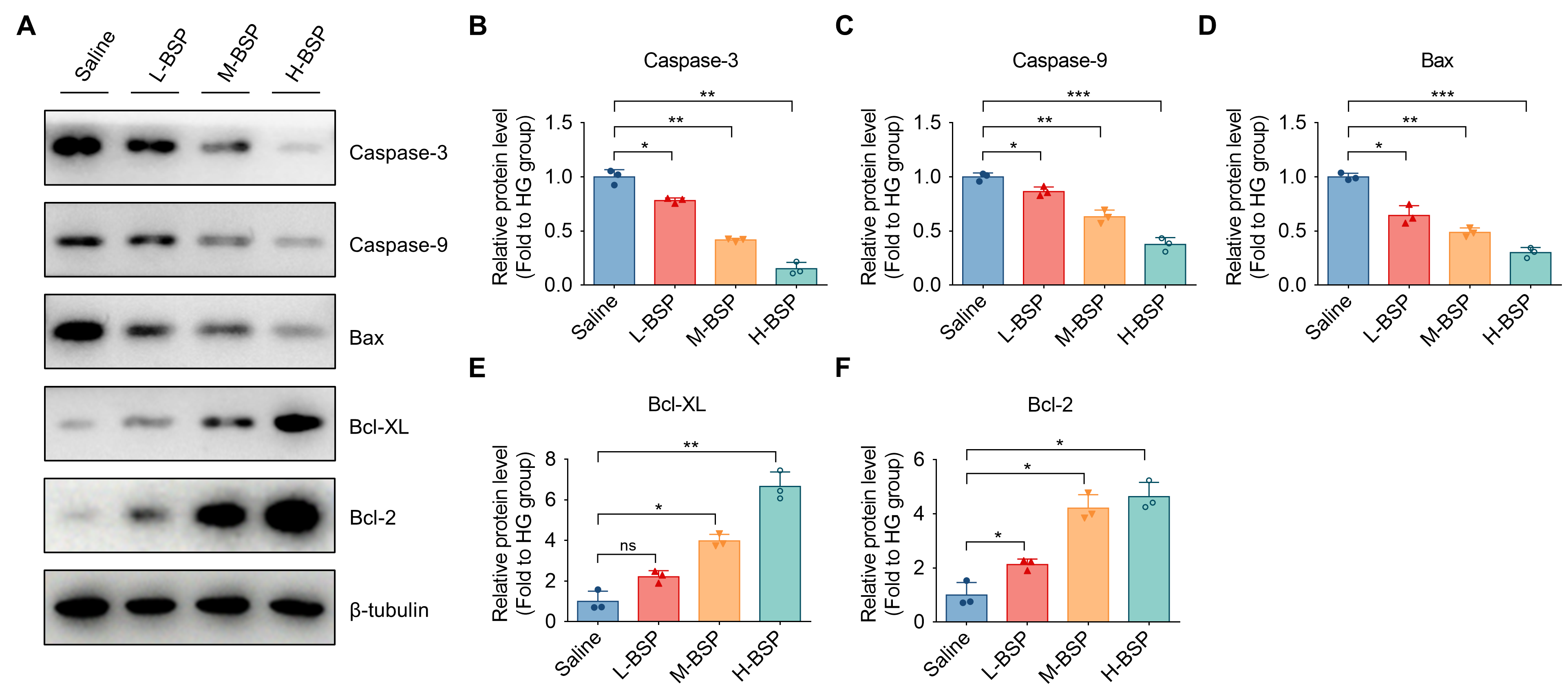


**Figure S4. BSP alleviated the apoptosis of L929 cells to accelerate diabetic wound healing in vivo.** (A) WB analysis of Caspase-3, Caspase-9, Bax, Bcl-XL and Bcl-2 of diabetic wounds tissue. (B-F) Quantitative analysis of Caspase-3, Caspase-9, Bax, Bcl-XL and Bcl-2 of diabetic wounds tissue. BSP: bletilla striata polysaccharide. Data are expressed as the mean ± SD (n = 3). **P<*0.05*, **P<*0.01, ****P*<0.001, vs the indicated groups. NS, no significant difference.


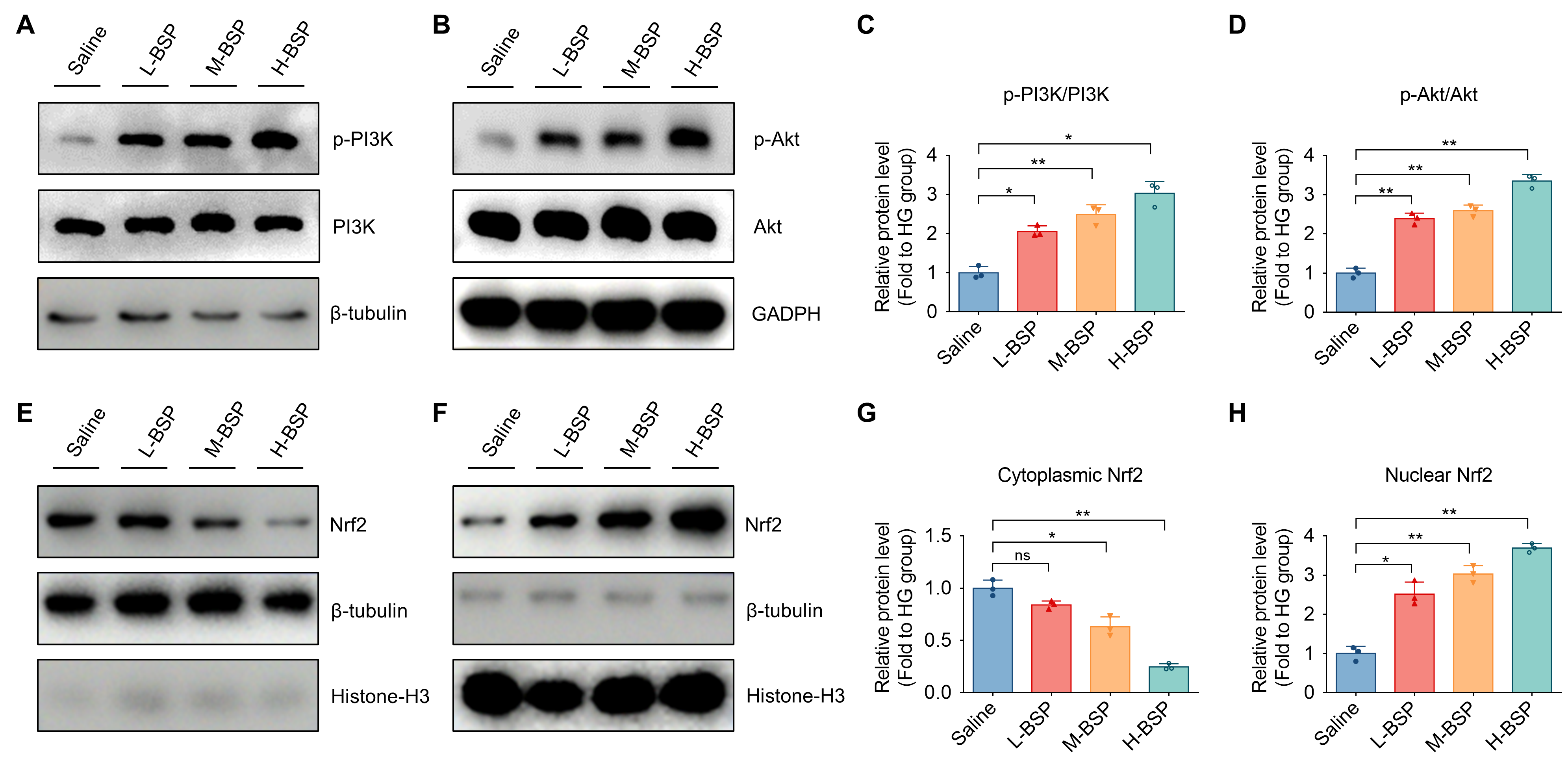
 **Figure S5. BSP improved the phosphorylation of PI3K/Akt and accelerated Nrf2 nuclear translocation in vivo.** (A and B) WB analysis of p-PI3K, PI3K, p-Akt, and Akt of diabetic wounds tissue. (C and D) Quantitative analysis of p-PI3K/ PI3K and p-Akt/Akt of diabetic wounds tissue. (E and F) WB analysis of cytoplasmic and nuclear Nrf2 of diabetic wounds tissue. (G and H) Quantitative analysis of cytoplasmic Nrf2 and nuclear Nrf2 of diabetic wounds tissue. BSP: bletilla striata polysaccharide. Data are expressed as the mean ± SD (n = 3). **P<*0.05*, **P<*0.01, vs the indicated groups. NS, no significant difference.
